# *Bacillus* populations restore amino acid metabolism in *Mesorhizobium* under saline–alkali stress to enhance nitrogen fixation efficiency

**DOI:** 10.1093/ismejo/wrag087

**Published:** 2026-04-14

**Authors:** Jiamin Ai, Mingxia Ren, Ziwei Hao, Yuru Zhai, Entao Wang, Zhenshan Deng, Zhefei Li

**Affiliations:** State Key Laboratory for Crop Stress Resistance and High-Efficiency Production, Shaanxi Key Laboratory of Agricultural and Environmental Microbiology, College of Life Sciences, Northwest A&F University, Yangling, Shaanxi 712100, China; College of Life Sciences, Yan’an University, Yan’an, Shaanxi 716000, China; College of Life Sciences, Yan’an University, Yan’an, Shaanxi 716000, China; College of Life Sciences, Yan’an University, Yan’an, Shaanxi 716000, China; State Key Laboratory for Crop Stress Resistance and High-Efficiency Production, Shaanxi Key Laboratory of Agricultural and Environmental Microbiology, College of Life Sciences, Northwest A&F University, Yangling, Shaanxi 712100, China; Departamento de Microbiología, Escuela Nacional de Ciencias Biológicas, Instituto Politécnico Nacional, Cd. México 11340, Mexico; College of Life Sciences, Yan’an University, Yan’an, Shaanxi 716000, China; State Key Laboratory for Crop Stress Resistance and High-Efficiency Production, Shaanxi Key Laboratory of Agricultural and Environmental Microbiology, College of Life Sciences, Northwest A&F University, Yangling, Shaanxi 712100, China

**Keywords:** *Sophora davidii*, succession, interaction, cross-feeding

## Abstract

The root nodules formed by rhizobia and leguminous plants are specialized structures for nitrogen fixation. However, a large number of non-rhizobial endophytes also coexist within the nodules, and their contribution to nitrogen fixation under abiotic stress conditions remains unclear. Here, using the wild leguminous shrub *Sophora davidii* as model system, we identified an important NRE (*Bacillus siamensis* BT-9-1) by analyzing keystone taxa within the bacterial cooccurrence network of root nodules. This strain could improve the survival of *Mesorhizobium metallidurans* YC-39 under saline–alkali stress. A mechanistic investigation revealed that the expression of *ilvA*, *ilvH*, and *ilvD* was downregulated, and the contents of (2S)-isopropylmalate and succinic acid decreased in *M. metallidurans* YC-39 under saline–alkali conditions, whereas *B. siamensis* BT-9-1 presented increased accumulation of these metabolites. These findings indicate that *B. siamensis* BT-9-1 cross-feeds *M. metallidurans* YC-39 with these metabolites, rescuing the compromised branched-chain amino acid synthesis pathway and the tricarboxylic acid cycle in saline–alkali environments. Eventually, coinoculation with *B. siamensis* BT-9-1 and *M. metallidurans* YC-39, along with (2S)-isopropylmalate and succinic acid supplementation, increased nitrogenase activity of the symbionts. Our study reveals a novel mechanism by which non-rhizobial endophyte *Bacillus* species enhances the growth and nitrogen fixation efficiency of *M. metallidurans* under saline–alkali stress through the delivery of key metabolites.

## Introduction

In nature, plants grow in environments rich in microorganisms, and engage in complex and dynamic interactions with pathogenic, symbiotic, and beneficial microbes [1]. As emphasized by recent studies, plants should no longer be considered standalone entities but rather as holobionts [2], comprising the host and its associated microbial communities. Within this framework, the microbial symbionts and host are functionally integrated, and play crucial roles in plant nutrient acquisition, growth regulation, and responses to biotic and abiotic stresses [3]. Building on this functional integration and long-term host–microbe coevolution [4], the concept of the “hologenome” has been proposed [5], suggesting that the host genome and microbiome together constitute a unified functional and evolutionary entity. Consequently, host–microbiota interactions are regarded as key determinants of symbiotic stability and environmental adaptability.

Plants are typically subjected to multiple environmental stressors, including drought, salinity, and nutrient limitation, which severely constrain plant growth. Although plants have evolved intrinsic stress-response strategies through hormonal regulation [6], metabolic reprogramming [7], and immune activation, increasing evidence indicates that these responses are strongly regulated by the microbial communities associated with plants [8]. Microorganisms typically increase plant stress resistance by improving nutrient availability, regulating plant hormone levels, and inducing systemic resistance [9]. However, environmental stressors may disrupt the metabolic capacity and growth of beneficial microbes, such as by limiting the synthesis of key metabolites [10]. Under these conditions, other members of the microbial community can provide metabolic compensation (e.g. cross-feeding), thereby restoring microbial function and synergistically alleviating plant stress. For example, *Bacillus velezensis* SQR9 cooperates with *Pseudomonas stutzeri* in the rhizosphere via cross-feeding interactions, resulting in enhanced plant growth and alleviation of salt stress. Cross-nutrient exchange among microbes is common in complex communities and plays a crucial role in shaping microbial community structures within plant tissues and regulating host health [11]. For example, thiamine auxotrophy of the plant-beneficial endophytic fungus *Serendipita indica* could be satisfied by the ubiquitous soil microorganism *Bacillus subtilis*, and this critical auxiliary trophic interaction has been shown to play an important role in the functioning of plant ecosystems [12]. Consequently, cross-feeding is increasingly recognized as a key ecological mechanism driving the functional stability of plant-associated microbial communities and host adaptability.

The legume-rhizobia symbiotic system represents a classic model of plant–microbe interactions [13]. Inside the nodules, the rhizobia differentiate into bacteroids, which convert N_2_ to NH₄^+^. This provides the host with available nitrogen, thereby enhancing plant growth and stress tolerance [14]. Therefore, biological nitrogen fixation (BNF) is a sustainable method for enhancing soil fertility and reducing dependence on chemical fertilizers. However, improper irrigation, excessive use of chemical fertilizers, and climate change have accelerated soil salinization worldwide [15]. Saline-alkali stress severely inhibits the growth of rhizobia, nodule formation and nitrogen fixation efficiency, thus limiting the beneficial effects of the legume-rhizobia symbiotic system on the soil. Recent studies have shown that, in addition to rhizobia, various non-rhizobia endophytes (NREs) colonize nodules and play important roles in enhancing symbiont nitrogen fixation efficiency [16–18]. *Bacillus* species are often found in soybean nodules, where they can interact with rhizobia, either positively or negatively affecting their growth [19]. For instance, *Bacillus cereus* can specifically promote the growth of *Sinorhizobium*, while alleviating the effects of saline–alkali conditions on nodulation [20]. However, the specific molecular mechanisms by which NREs assist rhizobia in resistance to saline–alkali stress remain unclear, particularly whether NREs can provide key metabolites to rhizobia under stress conditions to achieve metabolic compensation, thereby maintaining their growth, nodulation, and symbiotic nitrogen fixation functions. Therefore, elucidating the interactions between NREs and rhizobia under saline–alkali conditions is essential for developing strategies to increase BNF efficiency and promote the growth of crops in salt-affected soils.

*S. davidii* is a pioneer species in the arid zone of the Loess Plateau in China and has important ecological value in terms of plant community succession, species diversity maintenance, and ecosystem maintenance [21]. To clarify the microbial interactions within the nodules of *S. davidii* and to elucidate the putative biotic interactions mediated by metabolic cross-feedings between key NREs and rhizobia, the nodules of *S. davidii* were collected at different developmental stages for analysis of the composition and function of the nodule-associated microbiota via 16S rRNA gene amplicon sequencing. Furthermore, the relationships between NREs and rhizobia were systematically explored under salt stress conditions. Finally, the mechanisms by which NREs enhance rhizobial fitness were elucidated on the basis of their ability to perform cross-feeding to alleviate saline–alkali stress.

## Materials and methods

### Collection of rhizosphere soil and nodule samples

Rhizosphere soils and nodules were collected in 2021 from wild-grown *S. davidii* (36°37′15″N, 109°22′8″E) in Yan’an City, Shaanxi Province, which is located in the Loess Hilly Region, an area of critical soil erosion in China, and is also saline–alkali land. The soil pH of the sampling site during different stages ranged from 8.22 to 8.47, and the electrical conductivity ranged from 1.81 to 2.35 dS/m, which is consistent with the international classification of mildly saline–alkali soils [22]. The samples were collected at three developmental stages: young nodules (small size, light brown colour, no BFN activity) were collected on April 9; active nodules (red–brown/red colour, BFN activity) were collected on June 20; and senescent nodules (brown colour, no BFN activity) were collected on August 20 [23]. The nodules used in this study were surface sterilized to ensure that the isolated bacteria were endophytes of the nodules. A detailed nodules surface-sterilization protocol is provided in Supplementary Material S1. To obtain biological replicates, we randomly marked three plots and harvested three *S. davidii* individuals at the same growth stage from each plot. A minimum distance of 3 m was maintained between individual plants to minimize plant-to-plant variation and ensure the representativeness of the samples. Rhizosphere soil and nodules were collected from each harvested plant by excavating parts of the root system, whereas bulk soil was collected from the adjacent root-free area (at least 10 cm away from the root zone) (Fig. S1A) [24]. After parts of the roots were carefully extracted from the soil, the nodules were excised from the roots with a scalpel as previously described [25], and the nodules were collected into sterile centrifuge tubes. To collect rhizosphere soil, the root system was first vigorously shaken to remove loosely attached bulk soil. The roots with tightly attached rhizosphere soil were then transferred into sterile 50 ml centrifuge tubes containing 25 ml of sterile 1× PBS buffer and transported to the laboratory. The tubes were subsequently vortexed to detach rhizosphere soil from the root surface. After vortexing, the root tissues were removed, and the remaining suspension was centrifuged at 8000 rpm for 7 min to pellet the soil particles. The resulting pellet was collected as the rhizosphere soil sample. Three nodules were collected from each plant, and a total of nine nodules were collected per sampling plot as a biological replicate. The sampled plants were labelled and left *in situ* to allow further growth and ensure consistent sampling across the three stages. In total, 27 samples were collected, including three compartments (nodules, rhizosphere soil, and bulk soil), three nodule development stages (young, active, and senescent stages). Each sample was divided into two aliquots: the first was transported to the laboratory in sterile plastic bags on ice for bacterial isolation, and the second was immediately frozen and stored at −80°C for DNA isolation. The air-dried and sieved (through a 2 mm mesh screen) soil was mixed with water at a 1: 2.5 ratio for the determination of soil pH and conductivity.

### Microbiome analysis

Total DNA was extracted separately from 0.5 g samples (fresh nodules, rhizosphere, and bulk soil) suing a TIANamp Soil DNA Kit (TIANGEN Biotech, Beijing, China) according to the manufacturer’s instructions. The V3-V4 region of the bacterial 16S rRNA genes were amplified using the barcoded primers 341F (5′-CCTAYGGGRBGCASCAG-3′) and 806R (5′-GGACTACNNGGGTATCTAAT-3′). Taxonomic affiliations of the unique amplified sequence variants (ASVs) were identified using the SILVA reference database (v138), and their relative abundances were calculated within the R environment (version: 4.4.1, http://www.r-project.org/). The detailed methods are provided in Supplementary Material S1. The Illumina sequence data for all the samples in this study were submitted to NCBI GenBank under BioProject PRJNA1144836.

### Interaction assay

To understand the inter associations among microbial taxa across different compartments, we integrated samples from all developmental stages and constructed co-occurrence networks for nodules, rhizosphere, and bulk soil separately. The pairwise Spearman correlation matrix was calculated from relative abundances at the genus level with the “corr.test” function of the "psych" package in R (v4.4.2). The *P* values were adjusted using the False Discovery Rate method. A threshold for Spearman’s rank correlation coefficient (ρ) greater than 0.9 or less than −0.9 (*P* < .05) was applied for network construction, and the network was visualized using Gephi. To simplify the nodule network further and to specifically examine the role of NREs, we extracted a subnetwork composed of shared genera—i.e. those present in all three nodule developmental stages. Nodes within this subnetwork were ranked using the hub index, thereby identifying keystone taxa critical for maintaining subnetwork integrity. The hub index is a metric we use to quantify the importance of specific nodes in a network, and the calculation is based on node degree and the HITS algorithm [26], which reflects the node's influence and centrality in the network [27]. Using Gephi’s statistical tools, it can easily compute hub scores.

**Figure 1:**
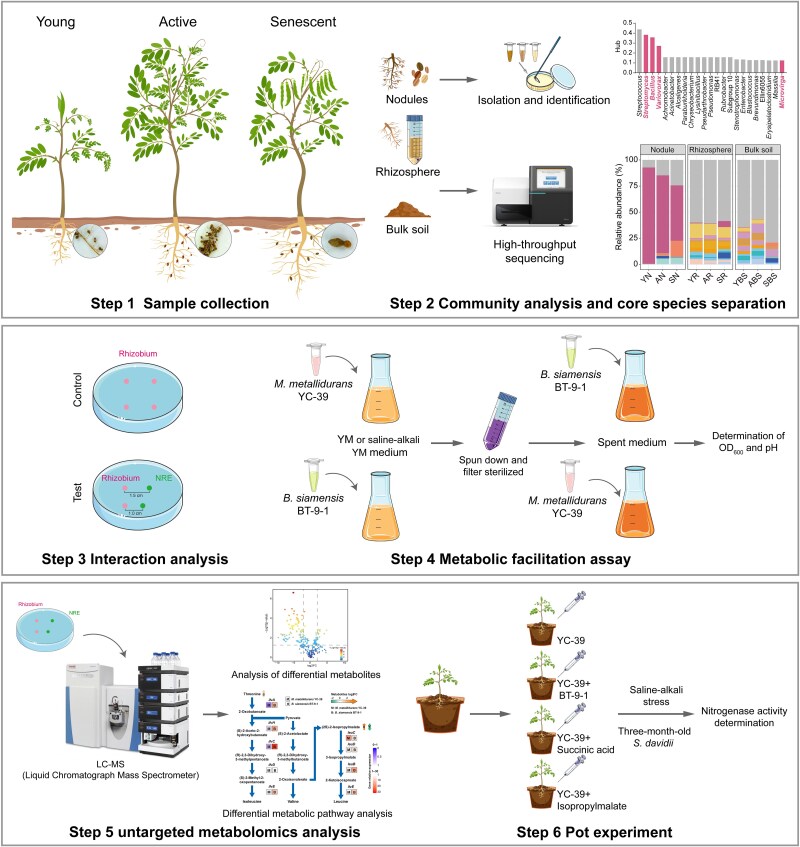
Flow charts of the nodule-associated bacterial community and metabolic cross-feeding between rhizobia and NREs. Step 1. Samples from different spaces (microhabitats) and times (nodules at three developmental stages) were collected. Step 2. The bacterial community compositions of the nodules, rhizosphere and bulk soils were analysed. The core nodule microorganisms were isolated and identified. Step 3. The interaction between the rhizobial and nonrhizobial species was analysed under various culture conditions. Step 4. Metabolic promotion between *M. metallidurans* YC-39 and *B. siamensis* BT-9-1. Step 5. Metabolomic analysis of *M. metallidurans* YC-39 and *B. siamensis* BT-9-1 under coculture conditions. Step 6. A pot experiment confirmed that the coapplication of metabolites and rhizobia enhanced nitrogenase activity.

The methods used for the isolation of symbiotic and NREs bacteria are detailed in Supplementary Material S1. The dominant symbiont, *Mesorhizobium* (*Mesorhizobium metallidurans* YC-39), was successfully isolated from the nodules. The isolated core strains were used as candidate strains for interaction analysis with rhizobium (*M. metallidurans* YC-39). Under normal conditions, candidate NREs and rhizobium strains were cultured in YM broth (YMB) for 3–4 days at 28°C with shaking at 180 rpm, and the cultures were adjusted to an optical density (OD) of 1.0 at 600 nm. Next, 2 μl aliquots of each candidate strain in suspension were inoculated at a 1.0-or 1.5-cm distance from the inoculation point of the rhizobium strain (*M. metallidurans* YC-39) on YM agar (YMA) plates (90 mm in diameter) (Fig. 1, step 3) [20]. The colony diameter of the rhizobium strain was calculated, and the interaction effect was evaluated by comparison with that of a single inoculation of rhizobium. The plates were incubated at 28°C for 4–7 days. Images were captured using an automatic colony analyser (Czone G6T, Shineso, China).

Interaction of strains under stress conditions: Using YMB as the basal medium, the growth of the isolates was tested at different pH values (4, 5, 6, 7, 8, 9, 10, and 11) and different salt concentrations (0, 5, 10, 20, 30, 40, 50, and 60 g/l, pH 7.0). Saline–alkali stress conditions (pH = 8.5, NaCl = 0.5%) allowed the growth of NREs, while significantly inhibiting the growth of the rhizobium strain. Subsequently, we employed the same experimental design as described above to explore the interaction between rhizobium and NREs. In this test, different concentrations of *M. metallidurans* YC-39 (OD_600_ = 0.5 and 1.0) were used. Exopolysaccharide (EPS) production and biofilm formation were measured as indicators of growth promotion or inhibition in response to microbial interactions [28]. The detailed methods are shown in Supplementary Material S1.

### Metabolic facilitation and chemotaxis assay

To further explore whether the interaction between *B. siamensis* BT-9-1 and *M. metallidurans* YC-39 is associated with metabolic facilitation, spent medium complementation assays and metabolite chemotaxis assays were conducted. Both the NREs and rhizobial isolates were individually inoculated in 20 ml of YMB and incubated at 28°C with shaking at 180 rpm. The optical density (OD_600_) was measured daily. After entering the exponential growth phase, the cell cultures were centrifuged (10 000 rpm, 4°C for 10 min), and the supernatants were filter-sterilized (Fig. 1, step 4). Each isolate was then inoculated into the supernatant of the other strain at a 1% (v/v) ratio and incubated for 4 days at 28°C with shaking at 180 rpm. The final OD_600_ and pH values were measured at the end of the incubation period.

A capillary assay was performed according to methods described previously [29, 30]. Briefly, the assay setup consisted of a 200 μl pipette tip and a 2 cm 25-gauge needle (Fig. S1). *M. metallidurans* YC-39 and *B. siamensis* BT-9-1 were cultured in YMB until the OD_600_ reached 1.0. The bacterial culture was subsequently centrifuged and resuspended in a new YMB or saline–alkali YMB. A 200 μl pipette tip was used as a chamber to hold 100 μl of *M. metallidurans* YC-39 cell suspension (~1 × 10^7^ cells). The mixture was attached to a 1 ml syringe containing 200 μl of *B. siamensis* BT-9-1 supernatant. After 3 h of incubation at room temperature, the needle syringe was removed from the bacterial suspension, and the content was diluted and plated on YMA plates. The cumulative number of bacteria in the capillary was calculated as the average colony-forming units (CFUs) obtained from three plates. The usability of the data were evaluated using the relative chemotaxis index (RCI), defined as the ratio of the cumulative number of bacteria entering the test capillary to the corresponding value in the negative control capillary [31].

### Metabolomics and transcriptome sequencing

To identify and disentangle the mechanism of cooperation, we performed untargeted metabolomics analysis of the *M. metallidurans* YC-39 and *B. siamensis* BT-9-1 coculture systems. Detailed metabolite extraction methods and HPLC-MS/MS conditions are shown in Supplementary Material S1. The UHPLC–MS/MS data were acquired in both positive and negative ionization modes, and the raw data were imported into CD 3.1 search software for processing. Each metabolite was identified by matching both its retention time and mass–charge ratio. The molecular formulae were then predicted from the molecular ion and fragment ions peaks and compared with the mzCloud (https://www.mzcloud.org/), mzVault, and Masslist databases, and finally, metabolite identification and relative quantification results were obtained. Metabolites were annotated using the KEGG, HMDB, and LIPIDMAPS databases. Differentially abundant metabolites were identified based on a fold change >2 and a *P* < 0.05. Pathway enrichment analyses were performed using the KEGG database based on the identified differentially abundant metabolites. Non-targeted metabolomics data have been uploaded to the MetaboLights database under Project MTBLS12739 and MTBLS12729.

For transcriptome sequencing, RNA was extracted using TRIzol reagent. Ribosomal RNA was removed from the total RNA and then precipitated with ethanol. After fragmentation, the cDNA was synthesized. Libraries of the amplified RNA from each pool were prepared using the Illumina protocol. Rawdata (raw reads) of the fastq format were first processed through fastp software. Rockhopper was used to identify novel genes, operon, TSS, TTS and Cisnatural antisense transcripts. Analysis of differentially expressed genes (DEGs) was conducted using the DESeq2 R package (1.42.0). The threshold of significant differential expression: *P* adj ≤ 0.05 and absolute fold change ≥1. The DEGs were subjected to KEGG annotation and enrichment analysis. The raw transcriptomic sequencing data have been submitted to NCBI, with the SRA accession numbers PRJNA1355509 and PRJNA1355517.

### Statistical analysis

Data are expressed as mean ± standard deviation (SD) derived from at least three independent biological replicates (n ≥ 3). Prior to statistical inference, the normality of data distribution and homogeneity of variance were assessed using Shapiro–Wilk and Levene’s tests, respectively. For multiple comparisons, a one-way analysis of variance (ANOVA) was performed, followed by Duncan's multiple range test to identify specific treatment effects. Pairwise comparisons between two groups were evaluated using Tukey test. ^*^, *P* < 0 .05; ^**^, *P* < 0 .01; ^***^, *P* < 0 .001. All the statistical analyses were conducted in the R environment (version: 4.4.1, http://www.r-project.org/). The Simpson index was used to assess the alpha diversity of the bacterial communities in the nodules, rhizosphere and bulk soils utilizing the “vegan” package in R. Principal coordinate analysis (PCoA) was conducted to evaluate the beta diversity via calculation of the unweighted UniFrac distance matrix [32]. Spearman correlations were computed between microorganisms using the “ggcor” package in R, with a Spearman's coefficient > 0.6 and a *P* < 0.05 considered thresholds for significant robust correlations [33]. Cytoscape 3.4.0 was employed to construct the profile network at the taxonomic level and to calculate network properties [34]. Figures were generated using the ggplot2 R package. Detailed statistical methods are provided in the figure legends.

## Results

### Successional dynamics of the microbial community in nodules and rhizosphere

**Figure 2:**
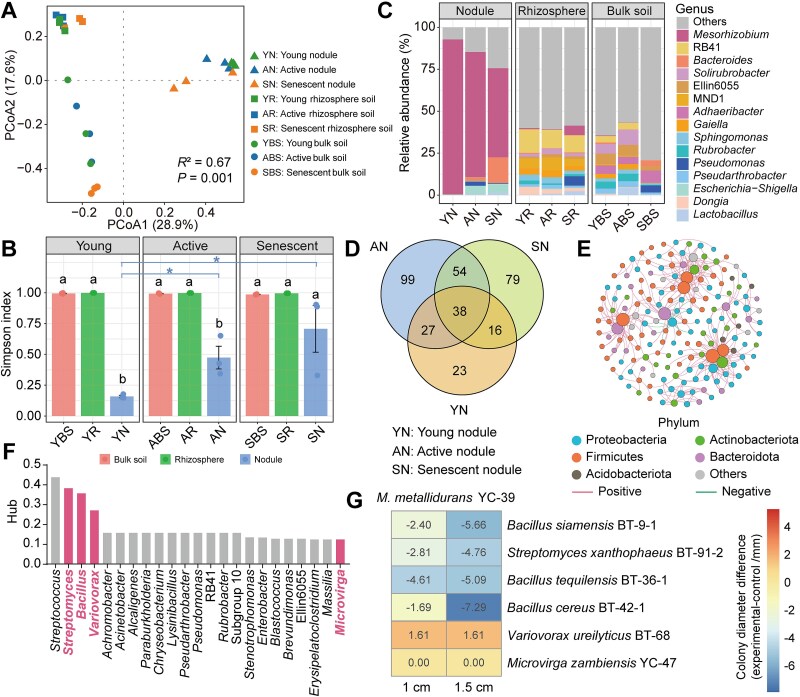
Comparison of bacterial community compositions. (A) PCoA of microbial communities on the basis of bray–Curtis distances. Differences among groups were evaluated using PERMANOVA (adonis2, 999 permutations). Y: Young stage; a: Active stage; S: Senescent stage; N: Nodules; R: Rhizosphere soil; BS: Bulk soil. Circles represent nodule samples, squares represent bulk soil samples, and triangles represent rhizosphere soil samples. (B) Simpson index of the bacterial community across various compartments and nodule developmental stages. Data are expressed as mean ± SD derived from at least three independent biological replicates (n = 3). Lowercase letters above the bars indicate significant differences (*P* < .05) between compartments in the three stages (one-way ANOVA). Asterisks indicate significant differences between three phases in the same compartment (Tukey test). ^*^, *P* < .05; ^**^, *P* < .01; ^***^, *P* < .001. (C) Taxonomic composition of bacterial communities at the genus level across developmental stages. (D) Common and unique genera at various developmental stages of nodules. (E) Cooccurrence network of common genera in the nodules. Each node in the network represents a genus and is coloured according to its phylum classification. (F) The top 23 genera in common genus co-occurrence networks were analysed via the hub index. (G) Interactions between core microorganisms and rhizobia. The colony diameter difference of rhizobium under single culture and coculture conditions was drawn by heat map.

PCoA based on Bray–Curtis distances revealed significant differences in bacterial community composition among the bulk soil, rhizosphere, and nodules during the three nodule development stages, with particular differences in the nodule senescence stage (Fig. 2A and Fig. S2A). In the three stages of nodule development, the Simpson index of the nodules was significantly lower than that of the rhizosphere and bulk soil (Fig. 2B), showing an increasing trend with nodules development. However, our previous results on the succession of diazotrophic community in *Sophora davidii* indicated that the α diversity of diazotrophic community within the nodules decreased as the nodules developed [35]. At the genus level, we also observed a declining trend in the relative abundance of the dominant symbiotic rhizobia, whereas the relative abundance of NREs significantly increased with nodule development (Fig. 2C). This suggests that the decrease in the relative abundance of rhizobia released certain ecological niches, and the increase in the relative abundance of NREs may be associated with the execution of specific ecological functions.

To investigate which bacteria were recruited by the nodules, the bacterial composition within the nodules was analysed. The results revealed that Proteobacteria gradually became enriched from the bulk soil to the rhizosphere and further to the root nodules. However, the relative abundance of Proteobacteria tended to decrease across the three stages of nodule development (Fig. S2B). In contrast, the relative abundances of Bacteroidota and Firmicutes significantly increased from the young to the senescent nodule stages (Fig. S2C). *Adhaeribacter* was the dominant genus in the bulk soil, while RB41 is the dominant genus in the rhizosphere. *Mesorhizobium* was absolutely dominant across all three nodule developmental stages but decreased in relative abundance over time (Fig. 2C). In addition, the relative abundance of 22 genera changed significantly across all three nodule developmental stages (Fig. S2D). Including *Mesorhizobium*, 38 genera were consistently detected across all three development stages of nodules. (Fig. 2D and Supplementary Material S2).

### Identification and isolation of core species within the nodules

At the genus level, the microbial community networks exhibited variations in the total number of nodes and links across the different samples. The bulk soil network presented the greatest number of nodes and links (Fig. S3A), whereas the nodule networks presented the lowest number. However, the nodule network presented the highest average degree and density (Fig. S3B), indicating that it was the most tightly connected microbial community. To further investigate the microbial interactions within the nodules, a subnetwork of 38 common genera was extracted from the nodule network (Fig. 2E). By analysing the hub indices of the nodes in the subnetwork related to 38 common genera, the core microbial genera with high connectivity in the subnetwork were identified. Further analysis revealed the top 23 core genera, which may have important impacts on the stability and function of the nodule microbial community (Fig. 2F).

To obtain symbiotic rhizobia and core NREs from the nodules of *S. davidii*, we isolated three rhizobia and 317 NREs from surface-sterilized nodules using YMA and LB medium. The analysis of bacterial community composition revealed that there were 13 rhizobia in the nodules, and the relative abundance of ASV_2 ranged from 30.1% to 92.5% in the three development stages of the nodules, indicating its dominant role in symbiotic nodulation (Supplementary Material S3). The isolated strain *Mesorhizobium metallidurans* YC-39 shared 98.8% sequence similarity with ASV_2 and presented the same changes in relative abundance as *Mesorhizobium* did (Fig. S4A). Furthermore, six of the 317 NREs were found to be aligned with the core genera identified within the subnetwork of common genera. These core genera included *Bacillus* (*B. tequilensis* BT-36-1, *B. siamensis* BT-9-1, *B. cereus* BT-42-1), *Streptomyces* (*S. xanthophaeus* BT-91-2), *Variovorax* (*Variovorax ureilyticus* BT-68), and *Microvirga* (*Microvirga zambiensis* YC-47) (Fig. 2G). These four core genera had the highest relative abundance in the nodules during the active stage of nodule development (Fig. S4B and Supplementary Material S4).

### Interaction between the rhizobium and NREs

**Figure 3:**
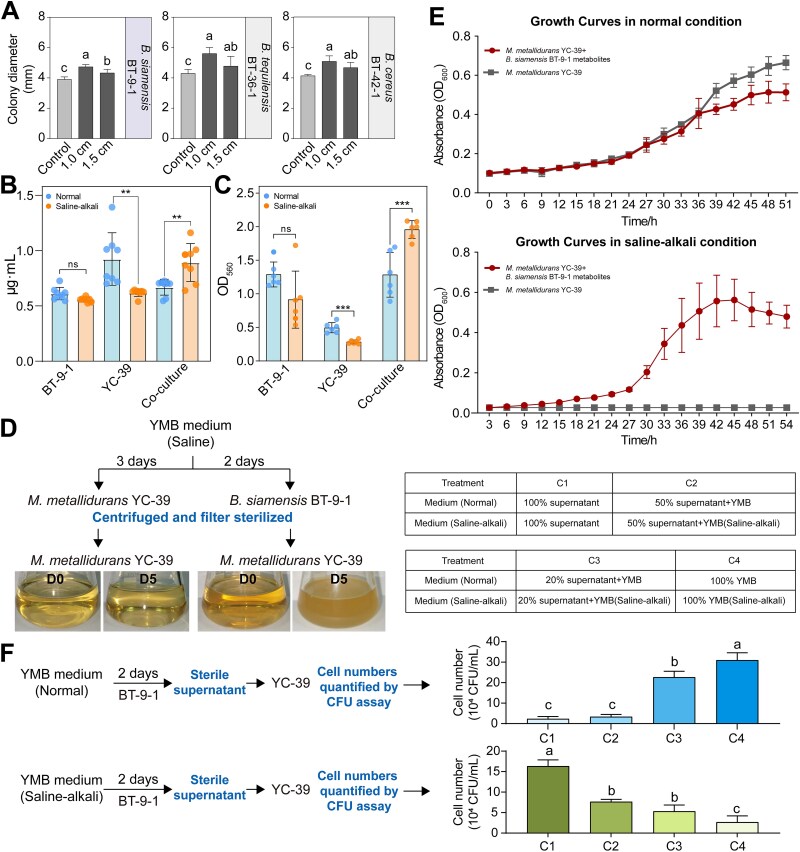
Interaction analysis of *Bacillus* and *Mesorhizobium*. (A) Colony diameters of *M. metallidurans* YC-39 after 4 days of coculture with different *Bacillus* species under saline–alkali stress; 1.0 and 1.5 cm represent the distance between *Bacillus* and *Mesorhizobium*. Lowercase letters above the bars indicate significant differences (*P* < 0.05) between the different treatments (one-way ANOVA). (B) EPS content by bacteria under various culture conditions. Lines represent the mean SD. Statistical significance was calculated using unpaired two-tailed *t*-tests. ^*^P < 0.05; ^**^P < 0.01 and ^***^P < 0.001. (C) Biofilm formation by bacteria under different culture conditions. (D) Schematic representation of the YMB spent medium experiments. Bacteria were independently grown in YMB medium for 3 days (*M. metallidurans* YC-39) or 2 days (*B. siamensis* BT-9-1), after which the cells were removed from the suspension. The filtrate was used as the growth medium for strain YC-39, D0: Day 0; D5: Day 5 after inoculation. (E) Growth curves of monocultures cultivated in pure YMB (grey); monocultures grown in metabolites from *B. siamensis* BT-9-1 (red) under normal conditions or saline–alkali stress. (F) Chemotaxis of *M. metallidurans* YC-39 to the metabolites of *B. siamensis* BT-9-1 under different culture conditions. The number of viable cells that entered the test capillary was quantified by a CFU assay after 3 h of culture. All the RCI values were greater than 2. Data are expressed as mean ± SD derived from at least three independent biological replicates (n = 8).

To investigate the interaction between NREs and the symbiotic rhizobium, six NREs and *M. metallidurans* YC-39 were cocultured on YMA plates (Normal conditions: pH = 7.5, and the NaCl content is 0.5 g/l). Under normal conditions, only *V. ureilyticus* BT-68 promoted the growth of *M. metallidurans* YC-39, while *M. zambiensis* YC-47 had no effect on its growth. The remaining four NREs exhibited inhibitory effects on the growth of *M. metallidurans* YC-39 in the pairwise co-culture in vitro (Fig. 2G). Given that the sampled plants were grown in saline–alkali soil, we hypothesized that these enriched bacteria might play a role in the saline–alkali tolerance of the symbiotic system. To test this hypothesis, we evaluated the interaction between the rhizobium and the six NREs under saline–alkali stress conditions. On the basis of the tolerance of *M. metallidurans* YC-39 to varying pH and NaCl concentrations, as well as the soil physicochemical properties (Fig. S5), we selected a pH of 8.5 and 0.5% NaCl as the saline–alkali stress conditions for testing the interactions between NREs and rhizobia. Under saline–alkali stress conditions, *B. siamensis* BT-9-1, *B. tequilensis* BT-36-1, and *B. cereus* BT-42-1 promoted the growth of *M. metallidurans* YC-39 on YMA plates, whereas the other strains did not affect its growth (Fig. 3A, Fig. S6). When the inoculation concentration of *M. metallidurans* YC-39 was reduced to OD_600_ = 0.5, only *B. siamensis* BT-9-1 was able to restore the growth of *M. metallidurans* YC-39 under saline–alkali stress conditions (Fig. S7), whereas the other two *Bacillus* strains failed to promote its growth at lower concentrations.

Previous studies have demonstrated that bacterial saline–alkali tolerance is closely related to EPS and biofilm formation [36]. Therefore, we further investigated EPS production and biofilm formation in *B. siamensis* BT-9-1 and *M. metallidurans* YC-39 under coculture conditions. Both *B. siamensis* BT-9-1 and *M. metallidurans* YC-39 exhibited reduced biofilm formation and EPS production under saline–alkali stress compared with normal culture conditions. Despite the inability to quantify individual contributions, coculturing these strains under saline–alkali stress resulted in a significant increase in total biofilm and EPS production (Fig. 3B, C).

### Effects of metabolites on the interaction between *B. siamensis* and *M. metallidurans*

In a subsequent study, we investigated the effects of metabolites produced by *B. siamensis* BT-9-1 on the growth of *M. metallidurans* YC-39. In the spent medium complementation assays under saline–alkali conditions, *M. metallidurans* YC-39 grew in the supernatant of *B. siamensis* BT-9-1 but not in its own supernatant or in YMB alone (Fig. 3D). The results demonstrated that the supernatant of *B. siamensis* BT-9-1 inhibited *M. metallidurans* YC-39 growth under normal conditions but promoted its growth under saline–alkali conditions (Fig. 3E). The chemotaxis assay results revealed that under normal conditions, the chemotaxis of *M. metallidurans* YC-39 to *B. siamensis* BT-9-1 metabolites decreased with increasing metabolite concentration, whereas it increased under saline–alkali conditions (Fig. 3F). These findings demonstrated that the addition of *B. siamensis* BT-9-1 supernatant promoted the growth of *M. metallidurans* YC-39 under saline–alkali stress.

### Metabolomic analysis of *B. siamensis* and *M. metallidurans*

**Figure 4:**
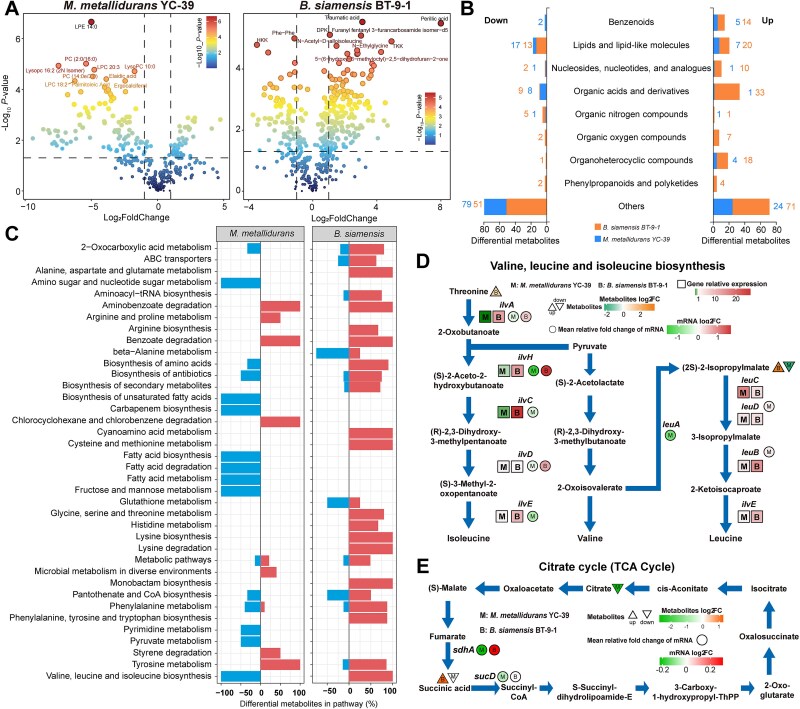
Metabolic responses of *M. metallidurans* YC-39 and *B. siamensis* BT-9-1. (A) Analysis of differentially abundant metabolites between the two strains. Differentially abundant metabolites were identified on the basis of a fold change >2 and a *P* < 0.05. (B) Classification of differentially abundant metabolites in *M. metallidurans* YC-39 and *B. siamensis* BT-9-1. (C) Differential metabolic pathways of *M. metallidurans* YC-39 and *B. siamensis* BT-9-1 under saline–alkali stress. (D) The relative fold change in the expression of genes involved in the branched-chain amino acid biosynthesis pathways, as examined by qRT–PCR. The colour of the square represents the mean relative fold change in gene expression in saline–alkali coculture compared with that under normal culture conditions (n = 6). The colour of the triangle represents the fold change in the expression of the substance under saline–alkali and normal culture conditions. The direction of the triangle represents the up- or downregulation of the expression of the substance under saline–alkali and normal culture conditions. The circle represents the folding changes of pathway genes expressed under saline–alkali and normal culture conditions in transcriptome analysis. The round color indicates that the gene is up-regulated or down-regulated under saline–alkali and normal culture conditions. (E) Differentially abundant metabolites annotated in the TCA cycle. The colour of the triangle represents the fold change in the expression of the substance under saline–alkali and normal culture conditions. The direction of the triangle represents the up- or downregulation of the expression of the substance under saline–alkali and normal culture conditions. The circle represents the folding changes of pathway genes expressed under saline–alkali and normal culture conditions in transcriptome analysis. The round color indicates that the gene is up-regulated or down-regulated under saline–alkali and normal culture conditions.

We conducted a metabolomic analysis to investigate which metabolites produced by strain *B. siamensis* BT-9-1 could alleviate saline–alkali stress in rhizobia. A total of 341 (annotated 156) and 447 (annotated 240) metabolites were identified in *M. metallidurans* YC-39 and *B. siamensis* BT-9-1, respectively (Fig. S8, Supplementary Material S5 and Supplementary Material S6). Among these metabolites, 43 were significantly increased and 108 were significantly decreased in *M. metallidurans* YC-39 in response to saline–alkali stress, with lipid and lipid-like molecules being the predominant categories. In *B. siamensis* BT-9-1, the levels of 178 and 85 molecules, including organic acids and their derivatives, as well as lipids and lipid-like molecules, were significantly increased and significantly decreased, respectively, in response to saline–alkali stress (Fig. 4A, B).

Using the KEGG database, we performed metabolic pathway annotation of the differentially abundant metabolites in the tested strains in response to saline–alkali stress. This analysis revealed 23 differential metabolic pathways in *M. metallidurans* YC-39, primarily involving amino acid metabolism or biosynthesis pathways, including valine, leucine, and isoleucine biosynthesis; tyrosine metabolism; phenylalanine metabolism; biosynthesis of amino acids; and arginine and proline metabolism (Fig. S8). These pathways encompassed a total of nine differentially abundant metabolites, three of which were upregulated and six of which were downregulated (Table S1). In *B. siamensis* BT-9-1 under saline–alkali stress, 48 differential metabolic pathways were identified. Among the top 25 metabolic pathways, 11 were amino acid pathways, including beta-alanine metabolism; tyrosine metabolism; phenylalanine metabolism; lysine degradation; biosynthesis of amino acids; phenylalanine, tyrosine, and tryptophan biosynthesis; cyanoamino acid metabolism; alanine, aspartate, and glutamate metabolism; glycine, serine, and threonine metabolism; cysteine and methionine metabolism; and valine, leucine, and isoleucine biosynthesis (Fig. S8). These pathways contained a total of 48 differentially abundant metabolites, 43 of which were upregulated and five of which were downregulated (Table S2).

Under saline–alkali conditions, valine, leucine and isoleucine biosynthesis were significantly increased in *B. siamensis* BT-9-1 but significantly reduced in *M. metallidurans* YC-39 (Fig. 4C). And the content of (2S)-isopropylmalate in this pathway was significantly decreased in *M. metallidurans* YC-39 and markedly increased in *B. siamensis* BT-9-1. Furthermore, transcriptomic analysis was performed to examine the expression of key genes involved in the valine, leucine, and isoleucine biosynthesis pathway. Intriguingly, the expression of *ilvA*, *ilvH ilvD,* and *leuA*, which are involved in this pathway, was significantly downregulated in *M. metallidurans* YC-39, whereas the expression of the remaining genes (*leuD* and *leuB*) was upregulated (Fig. 4D and Supplementary Material S7). The expression of all genes involved in this pathway was further validated by qRT-PCR. The results showed that the genes associated with the synthesis of the differential metabolite (2S)-isopropylmalate were significantly downregulated in *M. metallidurans* YC-39 but upregulated in *B. siamensis* BT-9-1. In contrast, the genes involved in the degradation of (2S)-isopropylmalate were upregulated in *M. metallidurans* YC-39. These results suggest that the reduced production of (2S)-isopropylmalate in *M. metallidurans* YC-39 might be compensated by *B. siamensis* BT-9-1. Specifically, *M. metallidurans* YC-39 utilizes intermediates produced by *B. siamensis* BT-9-1 for the synthesis of branched-chain amino acids (BCAAs), thereby compensating for defects in the branched-chain amino acid synthesis function of rhizobia under saline–alkali stress conditions. This mechanism ensures the minimum requirement of BCAAs needed for survival. These findings demonstrate that metabolic cross-feeding interactions may occur between rhizobia and NREs.

In addition, the tricarboxylic acid (TCA) cycle was significantly upregulated in *B. siamensis* BT-9-1 under saline–alkali conditions, and the content of the intermediate succinic acid was significantly increased (Fig. 4E). Conversely, pyruvate metabolism, which serves as a vital link between glycolysis and the TCA cycle, was downregulated in *M. metallidurans* YC-39. Additionally, the contents of TCA cycle intermediates, including succinic acid and citrate, were reduced in *M. metallidurans* YC-39. The above results indicate that *M. metallidurans* YC-39 may utilize the TCA cycle intermediates released into the environment by *B. siamensis* BT-9-1 to meet its energy requirements.

### Coinoculation alleviates saline–alkali stress in the symbiotic system

We conducted pot experiments to evaluate the effects of inoculation with *M. metallidurans* YC-39 or *B. siamensis* BT-9-1 on plant physiological indices under saline–alkali stress (Fig. S9). Single inoculation of either strain resulted in a significant increase in superoxide dismutase (SOD) activity, soil urease activity, proline (Pro) content, and chlorophyll (a and b) levels while reducing malondialdehyde (MDA) accumulation. Furthermore, coinoculation with the two strains had a cumulative effect, as evidenced by significantly greater values for SOD, soil urease, Pro, and chlorophyll (*P* < 0.05) and significantly lower MDA levels than single inoculation. But no significant changes in soluble protein (SP) content were observed across all inoculation treatments. However, a significant increase in the soluble sugar (SS) content was observed only in the coinoculation treatment group compared with the control group. These results indicate that the combined inoculation of *M. metallidurans* YC-39 and *B. siamensis* BT-9-1 had a greater protective effect against saline–alkali stress than did the individual inoculation.

### Addition of differentially abundant metabolites improved the nodulation and nitrogen fixation of *S. davidii* under saline–alkali stress

**Figure 5:**
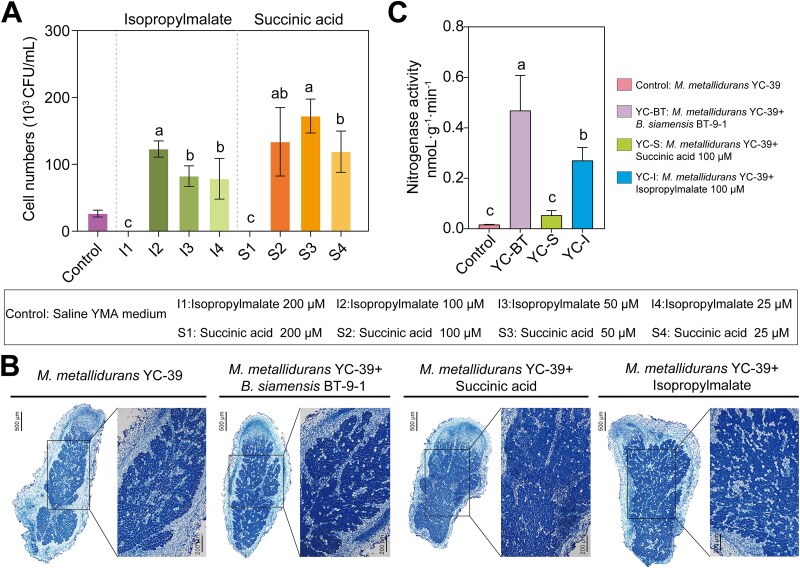
Effects of isopropylmalate and succinic acid on growth and nitrogen fixation in  *M. metallidurans*. (A) The addition of isopropylmalate and succinic acid mitigated saline–alkali stress in *M. metallidurans* YC-39. Data are expressed as mean ± SD derived from at least three independent biological replicates (n = 4). Lowercase letters above the bars indicate significant differences (*P* < 0.05) between the different treatments (one-way ANOVA). (B) Root nodules slices showing nodules sections subjected to different treatments. Scale bar = 500 μm. (C) The addition of differentially abundant metabolites increased the nitrogen fixation capacity of nodules under saline–alkali stress.

Metabolomic analysis revealed that *B. siamensis* BT-9-1 alleviated saline–alkali stress in *M. metallidurans* YC-39 by producing (2S)-isopropylmalate and succinic acid under coculture conditions. To further validate these findings, the effects of various concentrations of (2S)-isopropylmalate and succinic acid on the growth of *M. metallidurans* YC-39 under saline–alkali stress were investigated. The results demonstrated that high concentrations (200 μM) of both metabolites inhibited the growth of *M. metallidurans* YC-39 (Fig. 5A). At 100 μM, these metabolites significantly increased the survival of *M. metallidurans* YC-39 under saline–alkali stress. The results of the pot experiments further revealed that the number of bacteroids in the nodules inoculated with *M. metallidurans* YC-39 alone was lower, whereas the area occupied by bacteroids in the nodules inoculated with *M. metallidurans* YC-39 and *B. siamensis* BT-9-1 or supplemented with two metabolites was greater (Fig. 5B). Additionally, compared with inoculation with *M. metallidurans* YC-39 alone, treatment with *B. siamensis* BT-9-1, (2S)-isopropylmalate, or succinic acid significantly increased nitrogenase activity in nodules under saline–alkali stress (Fig. 5C). These findings indicate that the ability of *M. metallidurans* YC-39 to synthesize (2S)-isopropylmalate and succinic acid was impaired under saline–alkali stress, whereas *B. siamensis* BT-9-1 remediated this deficiency through metabolic cross-feeding, thereby increasing the nitrogen fixation capacity of root nodules and promoting the growth of host plants. These findings, from holobiont perspective, highlight that the adaptation of legume–rhizobium system to stress is the result of a metabolic dialogue between host plants and their associated microbial partners.

## Discussion

### Succession of nodule-associated bacterial communities

Root nodules are specialized nitrogen-fixing organs formed through the symbiosis between leguminous plants and rhizobia. In addition to rhizobia, a significant number of non-rhizobial endophytes (NREs) coexist within the nodules. These symbiotic bacteria may play an important role in enhancing the stress tolerance of rhizobia and improving their nitrogen fixation function [37]. Previous studies have primarily focused on the promotion of nodulation efficiency and plant growth by inoculating NREs. However, the mechanisms by which NREs assist symbionts in enhancing nitrogen fixation efficiency under abiotic stress conditions remain poorly understood. Our study reveals a novel mechanism by which NREs enhances the nitrogen fixation of rhizobia under saline–alkali stress through the delivery of key metabolites, highlighting their potential for enhancing rhizobium resilience in adverse environmental conditions. Moreover, these findings reinforce the holobiont perspective, in which the plant and its associated microbes act as an integrated functional unit, whose adaptive capacity emerges from context-dependent microbial interactions [5, 38]. As a pioneer plant, *S. davidii* grows extensively in the marginal soils of the Loess Plateau in China, and previous studies have shown that its dominant symbiont is *Mesorhizobium* [39, 40]. In this study, we investigated the dynamics of the bacterial community in the nodules of *S. davidii* and explored the interactions between *Mesorhizobium* and NREs within these nodules.

During the development of nodules, the relative abundance of *Mesorhizobium* exhibits a decreasing trend, while the α-diversity index within the nodules shows an increasing trend. Within nodules, rhizobia differentiate into functional bacteroids to adapt to nitrogen fixation. This transformation is accompanied by an increase in nitrogenase activity, but their metabolism and proliferative capacity is also limited [41]. Under abiotic stress conditions, the growth of rhizobia is inhibited, leading to a reduced proliferation rate and the release of ecological niches [42]. This allows for the growth and proliferation of NREs, resulting in an increase in their relative abundance. This is reflected in the significant increase in both the α diversity index and the relative abundance of NREs during the active stage. Increasing evidence suggests that NREs contribute to enhancing nitrogen fixation efficiency in nodules under saline–alkali stress conditions [20]. In this study, the peak relative abundance of *Bacillus* and the highest soil pH and conductivity were observed during the active stage. Experimental results also indicate that *B. siamensis* BT-9-1 significantly enhanced the colonization and nitrogenase activity of *M. metallidurans* YC-39 in root nodules under saline–alkali stress. It indicated that NREs in nodules are crucial for enhancing the nitrogen-fixing ability of symbionts under saline–alkali stress. In the final stage, the plant transitions from vegetative to reproductive growth. At this point, the plant reduces or ceases supplying carbon sources to the nodules, leading to nodules decay. Finally, some nodules began to fragment, and the nodule’s endophytes flowed out, which led to an increase in the relative abundance of *Mesorhizobium* in the rhizosphere. In summary, our study not only identifies specific functional hub microbes but also highlights the dynamic succession and ecological roles of NREs under saline–alkali stress.

Studies have shown that host plants selectively influence the structure and interactions of their related microbiota through “hub” microbes in the network and alter the adaptation of the host [43–45]. A study on soil community coalescence demonstrated that *Nitrospirillum* and *Microvirga* act as keystone nodes in rhizosphere microbial networks, where they enhance intra-community cooperation (e.g. via positive cohesion), thereby facilitating the proliferation of other microorganisms, increasing community stability, and ultimately promoting plant health and biomass [46]. Our study investigated the adaptive mechanisms of the legume–rhizobium symbiotic system under saline–alkali stress. We successfully isolated five “hub” microbes from nodules. Among them, *Bacillus* promoted the growth of *Mesorhizobium* under saline–alkali conditions and alleviated saline–alkali stress in plants, thereby enhancing the overall adaptive capacity of the holobiont. Previous studies have shown that *Bacillus* strains affect soybean nodulation and alleviate saline–alkali stress [20, 47]. In addition, the stress gradient hypothesis suggests that interspecific cooperative and competitive interactions shift along a nonbiological stress gradient, with cooperation dominating under high-stress conditions and competition increasing under low-stress conditions [48]. This hypothesis explains why *Bacillus* species inhibit the growth of rhizobia under normal conditions but promote their growth under saline–alkali stress. These findings suggest that the recruitment of *Bacillus* to nodules is closely related to the saline–alkali tolerance of the symbiosomes.

**Figure 6:**
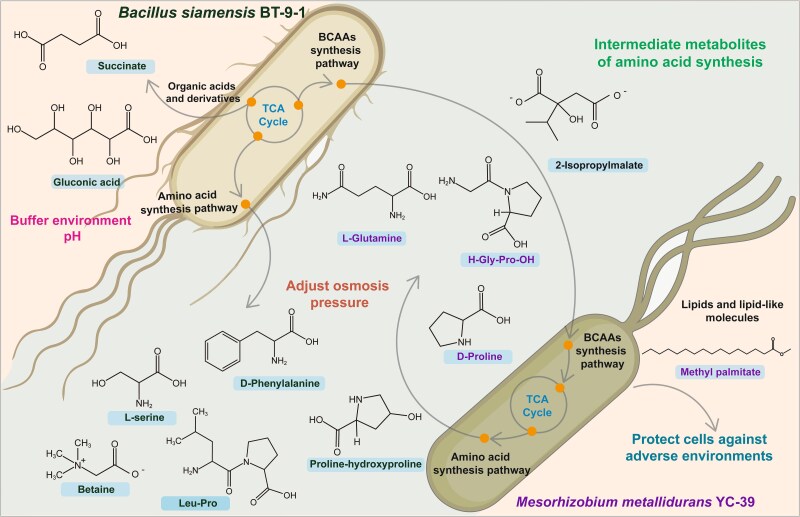
Schematic diagram of cross-feeding between *M. metallidurans* YC-39 and *B. siamensis* BT-9-1 on the basis of the results of the in vitro experiments. Under saline–alkali stress, the branched-chain amino acid synthesis (BCAA) pathway and the tricarboxylic acid (TCA) cycle in *M. metallidurans* YC-39 were inhibited, whereas these pathways were significantly upregulated in *B. siamensis* BT-9-1. Specifically, *M. metallidurans* YC-39 utilizes the intermediate product (2S)-isopropylmalate produced by *B. siamensis* BT-9-1 to synthesize BCAAs, thereby compensating for the reduced BCAA production under stress. Additionally, the energy demands of *M. metallidurans* YC-39 are satisfied by the use of TCA cycle intermediates released into the environment by *B. siamensis* BT-9-1, thereby improving its viability in saline–alkali environments. In addition, *B. siamensis* BT-9-1 can produce certain substances that help regulate osmotic pressure, enabling it to better adapt to saline–alkali stress.

### Metabolic interaction between *M. metallidurans* YC-39 and *B. siamensis* BT-9-1

Synergistic biofilm formation is a hallmark of plant-beneficial microbes. The multispecies biofilm lifestyle promotes the emergence of intrinsic community characteristics, such as enhanced tolerance to antimicrobial drugs, horizontal gene transfer, and the sharing of public goods [49–51]. Our study demonstrated that saline–alkali stress significantly reduced the biofilm formation of *B. siamensis* BT-9-1 and *M. metallidurans* YC-39 in monoculture but significantly increased in co-culture. Previous studies have shown that in the *B. subtilis*–*Pantoea agglomerans* interaction, both species contribute matrix components to the cocultured biofilm structure, with *B. subtilis* producing the matrix protein TasA and *P. agglomerans* producing EPSs [52]. When *M. metallidurans* YC-39 and *B. siamensis* BT-9-1 were cocultured under saline–alkali conditions, an increase in EPS production was detected for rhizobia. EPS play a critical role in rhizobium–legume symbiosis. EPS production can reduce direct bacterial–host cell contact, attenuate the host plant's defence response, and promote bacterial infection, metastasis, and colonization [53]. Furthermore, studies of EPS-deficient *Mesorhizobium alhagi* mutants have highlighted the importance of EPS production in stress adaptation [54]. Overall, these findings suggest that the synergistic formation of biofilms and EPS facilitates the adaptation of *M. metallidurans* YC-39 and *B. siamensis* BT-9-1 to saline–alkali conditions.

Microorganisms change the environment by absorbing resources and excreting metabolites, thereby affecting their own growth and that of other microorganisms [55]. A common environmental change is the modification of pH. For example, *Bacillus* sp. BP-3 releases pyruvic acid into the environment, leading to acidification and eventual self-limitation; however, this effect can be mitigated in coculture with *Delftia* sp. DT-2, as the latter utilizes pyruvic acid to meet its energy requirements, thereby preventing excessive acidification [55]. In our study, inoculation with *B. siamensis* BT-9-1 under saline–alkali stress reduced the pH value of the medium from the initial value of 8.5 to 7.91. In addition, metabolome analysis revealed that *B. siamensis* BT-9-1 significantly increased the production of acidic substances, such as succinic acid and gluconic acid, under saline–alkali stress conditions, thereby reducing the pH of the medium and alleviating saline–alkali stress in *M. metallidurans* YC-39. Additionally, the TCA cycle was significantly upregulated in *B. siamensis* BT-9-1 under saline–alkali stress, with succinate levels notably increasing. In *M. metallidurans* YC-39, the downregulation of TCA cycle intermediates such as citrate and succinate suggests inhibited steps within the cycle, potentially impairing energy production. Additionally, pyruvate metabolism, which serves as a critical link between glycolysis and the TCA cycle, is significantly downregulated, further exacerbating metabolic energy deficits. Since nitrogen fixation is an energy-intensive process that relies heavily on cellular energy systems, the downregulation of the TCA cycle and pyruvate metabolism in *M. metallidurans* YC-39 may lead to an insufficient energy supply, thereby compromising its nitrogen-fixing capacity. Therefore, under coculture conditions, *M. metallidurans* YC-39 may utilize TCA cycle intermediates released by *B. siamensis* BT-9-1 into the environment to meet its energy requirements and prevent continuous environmental acidification.

### *M. metallidurans* YC-39 and *B. siamensis* BT-9-1 cross-feeding via branched-chain amino acid synthesis pathways

The resource competition and metabolic cross-feeding of more than 800 microbial communities from diverse habitats were systematically investigated by named the 'species metabolic interaction analysis' metabolic modeling method, indicating that metabolic exchanges are widespread in natural communities [11]. The metabolic dissimilarity between the donor and recipient genotypes is a major determinant of the establishment of cross-feeding interactions between two bacterial strains [56]. In the present study, the differentially abundant metabolites of *B. siamensis* BT-9-1 were predominantly organic acids and their derivatives, followed by lipid-like molecules. The differentially abundant metabolites of *M. metallidurans* YC-39 were mainly lipid-like molecules. Therefore, the metabolic differences between *B. siamensis* BT-9-1 and *M. metallidurans* YC-39 lay the foundation for their interaction. Previous studies have shown that biosynthetically costly amino acids, including methionine, lysine, isoleucine, arginine, and aromatics, tend to promote stronger cooperative interactions than do amino acids, which are less expensive to produce. Furthermore, cells sharing common intermediates along branching pathways could yield more synergistic growth [57]. The metabolomics, transcriptomic, and qRT-PCR results of this study provide evidence for the possibility of cross-feeding via BCAA synthesis between *M. metallidurans* YC-39 and *B. siamensis* BT-9-1. The transcriptomic and qRT-PCR results indicated the expression of *ilvA*, *ilvH ilvD,* and *leuA*, which are involved in BCAA synthesis, was significantly downregulated in *M. metallidurans* YC-39, whereas the expression of the remaining genes (*leuD* and *leuB*) was upregulated (Fig. 4D). The content of (2S)-isopropylmalate was significantly reduced in *M. metallidurans* YC-39 and significantly increased in *B. siamensis* BT-9-1. It indicates that the intermediate products from *B. siamensis* BT-9-1's BCAA synthesis pathway compensate for the defects in the branched-chain amino acid synthesis function of rhizobia under saline–alkali stress conditions*.* In addition, transcriptomic analysis revealed that among the annotated genes of the two strains (excluding those classified as “Global and overview maps”), Membrane transport and Amino acid metabolism were the two significantly enriched categories. The genes related to ABC transporters and the Bacterial secretion system were upregulated in *M. metallidurans* YC-39, indicating that under saline–alkali stress, active exchange of substances and reprogramming of amino acid metabolism occurred between the two strains (Fig. S10). These findings align with the findings of Sun et al. [30], who reported that in the *B. velezensis* SQR9–*P. stutzeri* XL272 interaction system, BCAA biosynthesis pathways are involved in syntrophic cooperation. The ability to synthesize BCAAs is critical for microbial survival under stress conditions; however, when the nutrients required are provided by coexisting species or other environmental sources, this ability may not be indispensable [30]. Comparative genomic analysis and metabolic modeling were conducted on over 6000 sequenced bacteria from various environments, and the results indicated that amino acid biosynthesis has been extensively optimized to reduce individual metabolic burdens and facilitate enhanced cross-feeding to support synergistic growth across the biosphere [57]. A recent experiment demonstrated that obligate cross-feeding could significantly expand the metabolic niche space of interacting bacterial populations, thus potentially positively selecting cross-feeding bacterial populations [58]. Here, metabolic cross-feeding may act as a mechanism by which NREs can facilitate the growth of rhizobia under saline–alkali stress conditions.

The results of the pot experiments demonstrated that the addition of (2S)-isopropylmalate significantly increased nitrogenase activity (Fig. 5B, C). Moreover, the introduction of metabolites enhanced rhizobium colonization within the nodules. Previous studies have shown that only BCAAs must be transported into bacteroids to support development and, consequently, nitrogen fixation [59]. In plants, bacteroids become symbiotic auxotrophs, and the transcriptional downregulation of BCAA biosynthesis depends on the plant-derived nutrient supply. Studies have shown that bacteroids with *aap bra* null mutations are fewer in number, smaller, and have a lower DNA content than the wild type [60]. Additionally, the widespread reduction in BCAA synthesis by bacteroids and their acceptance of some or all of these amino acids from plants have been observed in French bean, alfalfa, soybean, and pea [61]. In this study, *B. siamensis* BT-9-1 supplied (2S)-isopropylmalate to *M. metallidurans* YC-39, alleviating the malnutrition of rhizobia under stress conditions. The addition of exogenous (2S)-isopropylmalate increased the number of bacteroids in the nodules and increased nitrogenase activity. These findings indicate that the deficiency of BCAAs in bacteroids occurs not only from plants but also from bacteria that are symbiotic with them. Therefore, our results showed that *B. siamensis* BT-9-1 provided (2S)-isopropylmalate and succinic acid to *M. metallidurans* YC-39, thereby increasing the colonization of *M. metallidurans* in nodules, enhancing nitrogen fixation by the symbiont, and promoting plant growth. However, the molecular mechanisms by how *Bacillus* modulates rhizobial colonization and nodule formation under saline–alkali stress have not yet been fully characterized. Additionally, the universality of this beneficial interaction across diverse legume hosts remains a subject for future research.

In summary, based on our research results, an interaction mechanism model was constructed (Fig. 6). *B. siamensis* BT-9-1 responds to stress by producing several organic acids and their derivatives, particularly amino acids, likely representing important mechanisms driving interactions between rhizobia and NREs under saline–alkali stress. In contrast, *M. metallidurans* YC-39, whose synthesis of organic acids and their derivatives is downregulated under saline–alkali stress, cannot buffer the environmental pH through acid production. However, it can benefit from the metabolites of *B. siamensis* BT-9-1 during coculture. Additionally, the significant upregulation of BCAA biosynthesis pathways in *B. siamensis* BT-9-1 resulted in the production of many intermediate metabolites, reducing the metabolic costs associated with these pathways in *M. metallidurans* YC-39 and partially compensating for essential amino acids. Thus, *M. metallidurans* YC-39 was able to grow and multiply under saline–alkali stress with the help of *B. siamensis* BT-9-1. To date, the interaction between *Bacillus* and rhizobia has been studied mostly via phenotypic analysis and amplicon sequencing, with limited knowledge about interspecies interactions at the molecular level. Our study investigated the role of NREs in nodule microecology and revealed the mechanism of interactions between rhizobia and NREs. This study provides a scientific basis for the application of NREs with good ecological adaptability.

## Conclusions

The present study demonstrated the spatial (microhabitat) and temporal (three developmental phases of the root nodules) bacterial succession associated with the nodules, rhizosphere and bulk soil of *S. davidii*. Under saline–alkali stress, *B. siamensis* BT-9-1 buffers the environmental pH by producing various organic acids and their derivatives and increases the levels of various amino acids and betaine to regulate osmotic pressure. In addition, the significant upregulation of branched-chain amino acid synthesis pathways in *B. siamensis* BT-9-1 results in the production of many intermediate metabolites, allowing *M. metallidurans* YC-39 to reduce the metabolic costs associated with these pathways and partially compensate for essential amino acids. In addition, *M. metallidurans* YC-39 may utilize the TCA cycle intermediates released into the environment by *B. siamensis* BT-9-1 to meet its energy requirements. Thus, with the help of *B. siamensis* BT-9-1, *M. metallidurans* YC-39 was able to grow and reproduce under saline–alkali stress. The combination of *B. siamensis* BT-9-1 and *M. metallidurans* YC-39 alleviated saline–alkali stress in the host plants. Taking together, the findings of our study reveal the mechanism of interactions between rhizobia and NREs.

## Supplementary Material

Figure_S1_wrag087

Figure_S2_wrag087

Figure_S3_wrag087

Figure_S4_wrag087

Figure_S5_wrag087

Figure_S6_wrag087

Figure_S7_wrag087

Figure_S8_wrag087

Figure_S9_wrag087

Figure_S10_wrag087

Supplementary_Tables_wrag087

Supplemental_Material_1_wrag087

Supplemental_Material_2_wrag087

Supplemental_Material_3_wrag087

Supplemental_Material_4_wrag087

Supplemental_Material_5_wrag087

Supplemental_Material_6_wrag087

Supplemental_Material_7_wrag087

## Data Availability

Community profiling data (V3–V4 16S rRNA gene amplicon sequences) is accessible through BioProject ID PRJNA1144836. All code for data processing and raw data, organized per manuscript figure are available for download from GitHub repository (https://github.com/ajmaaa/Mesorhizobium-cross-feeding). Non-targeted metabolomics data have been uploaded to the MetaboLights database under Project MTBLS12739 and MTBLS12729. The raw transcriptomic sequencing data have been submitted to NCBI, with the SRA accession numbers PRJNA1355509 and PRJNA1355517.
