# Supplementary figures and images for: Bacillus populations restore amino acid metabolism in Mesorhizobium under saline–alkali stress to enhance nitrogen fixation efficiency
Source: ISME J. 2026 Apr 14;20(1):wrag087. doi: 10.1093/ismejo/wrag087 (PMC13155107; doi:10.1093/ismejo/wrag087)

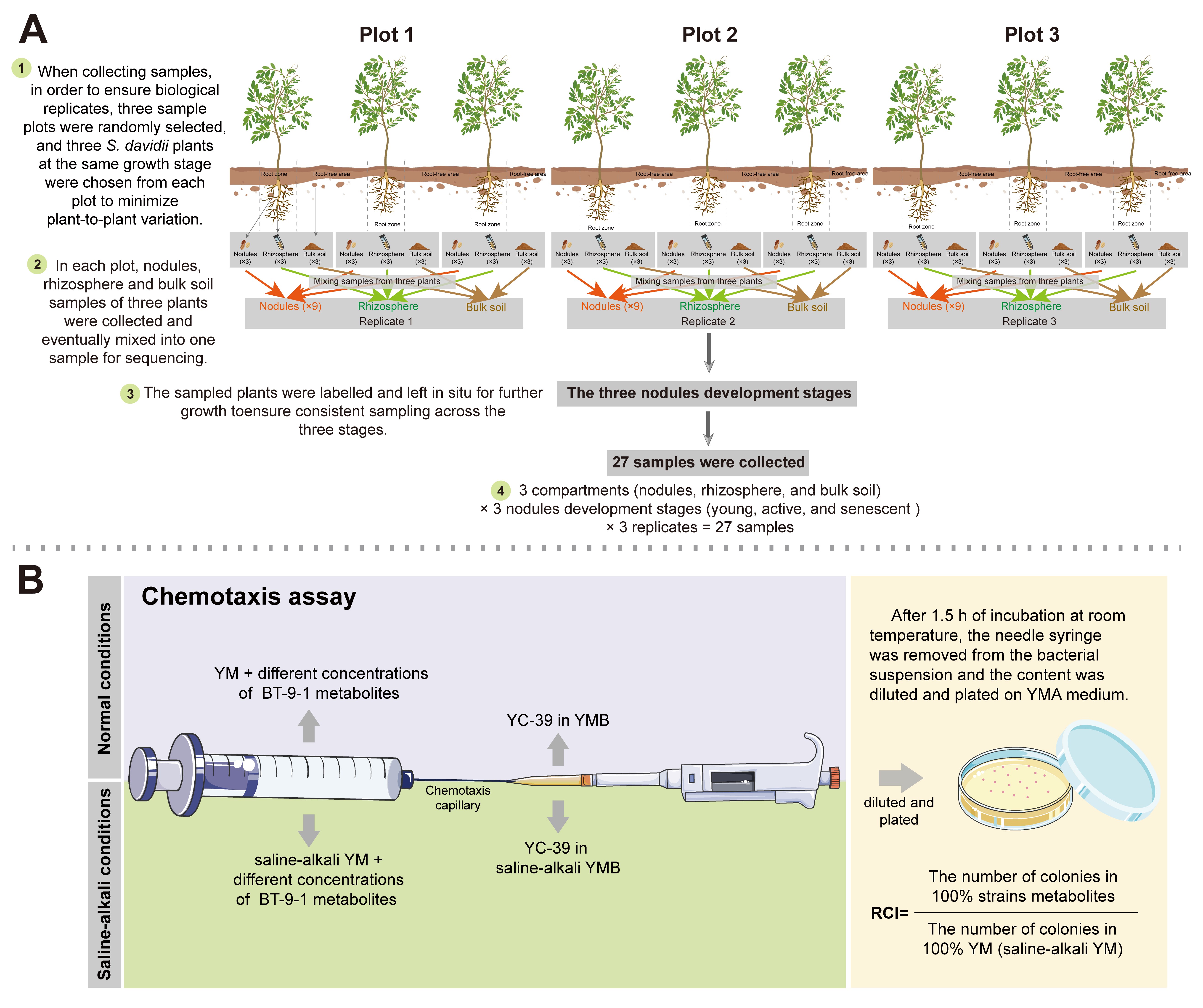

Supplement: Figure_S1_wrag087 [file figure_s1_wrag087.jpeg]

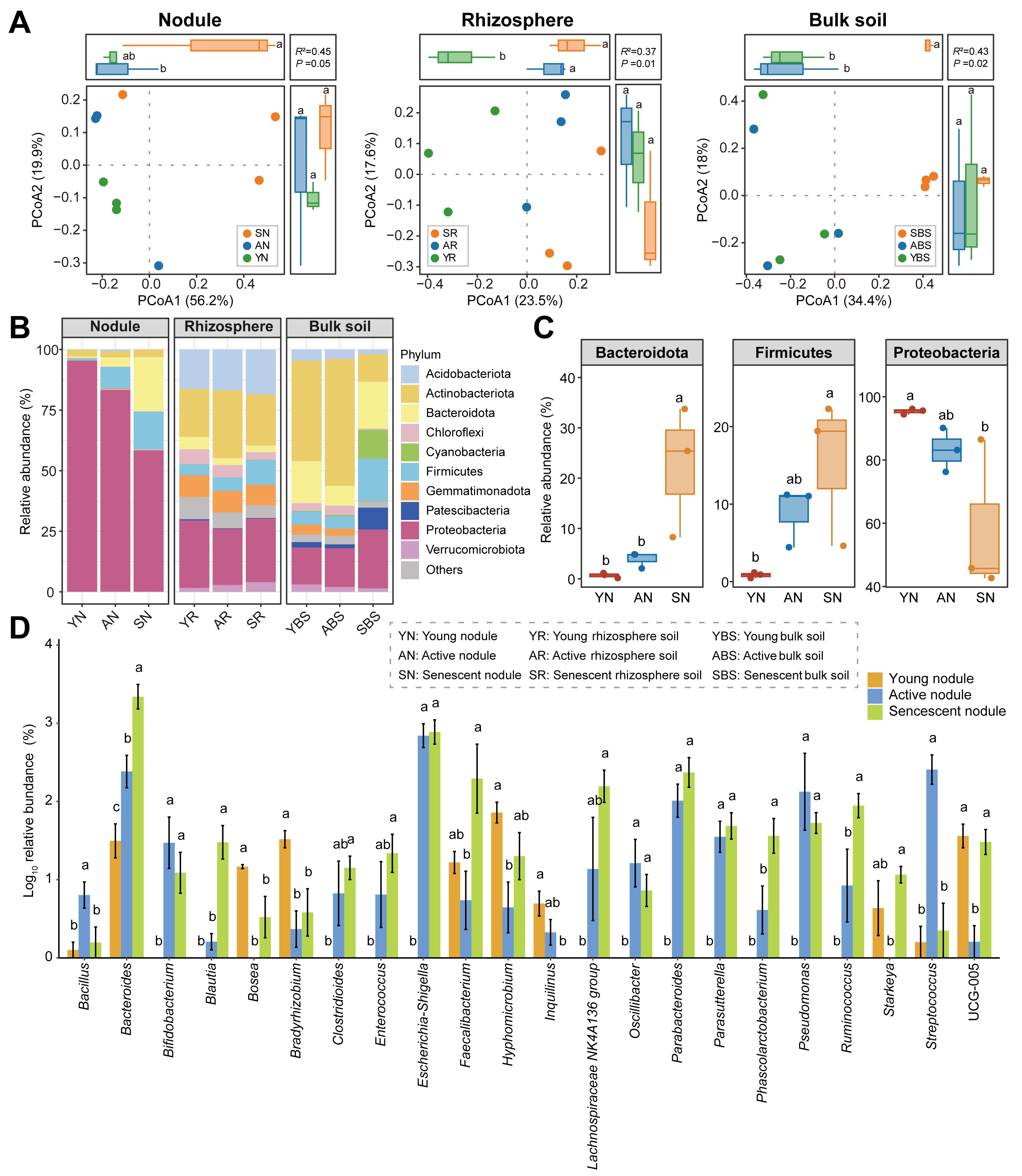

Supplement: Figure_S2_wrag087 [file figure_s2_wrag087.jpeg]

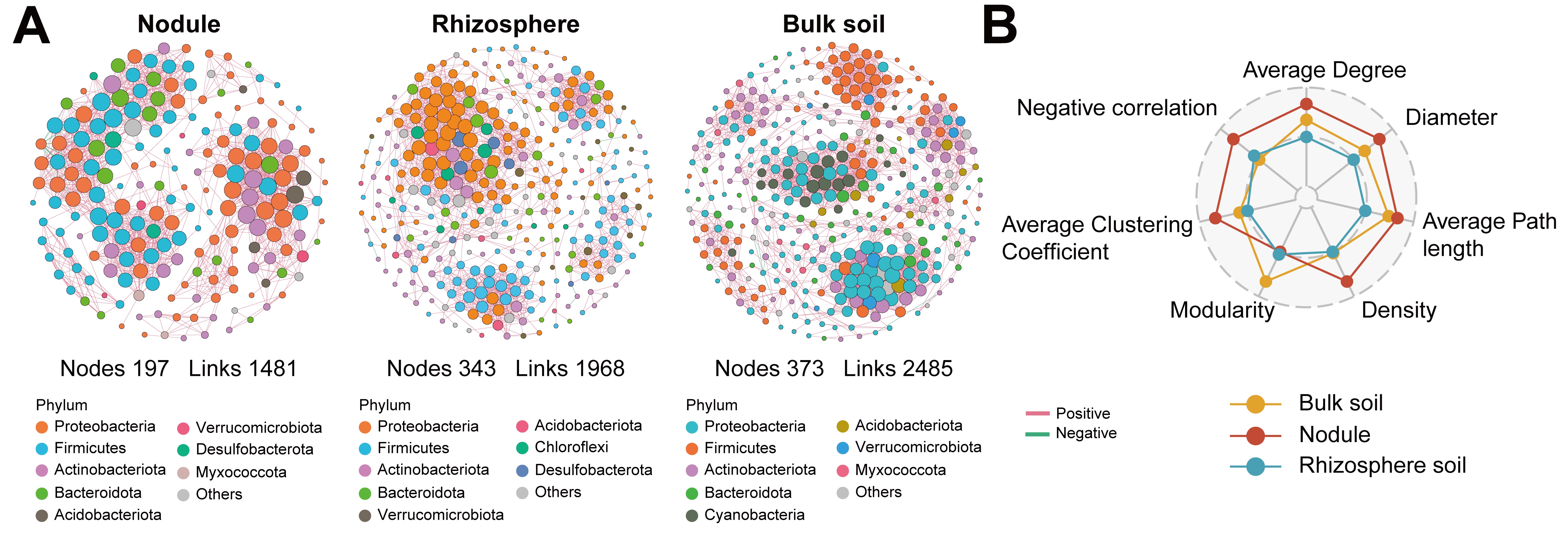

Supplement: Figure_S3_wrag087 [file figure_s3_wrag087.jpeg]

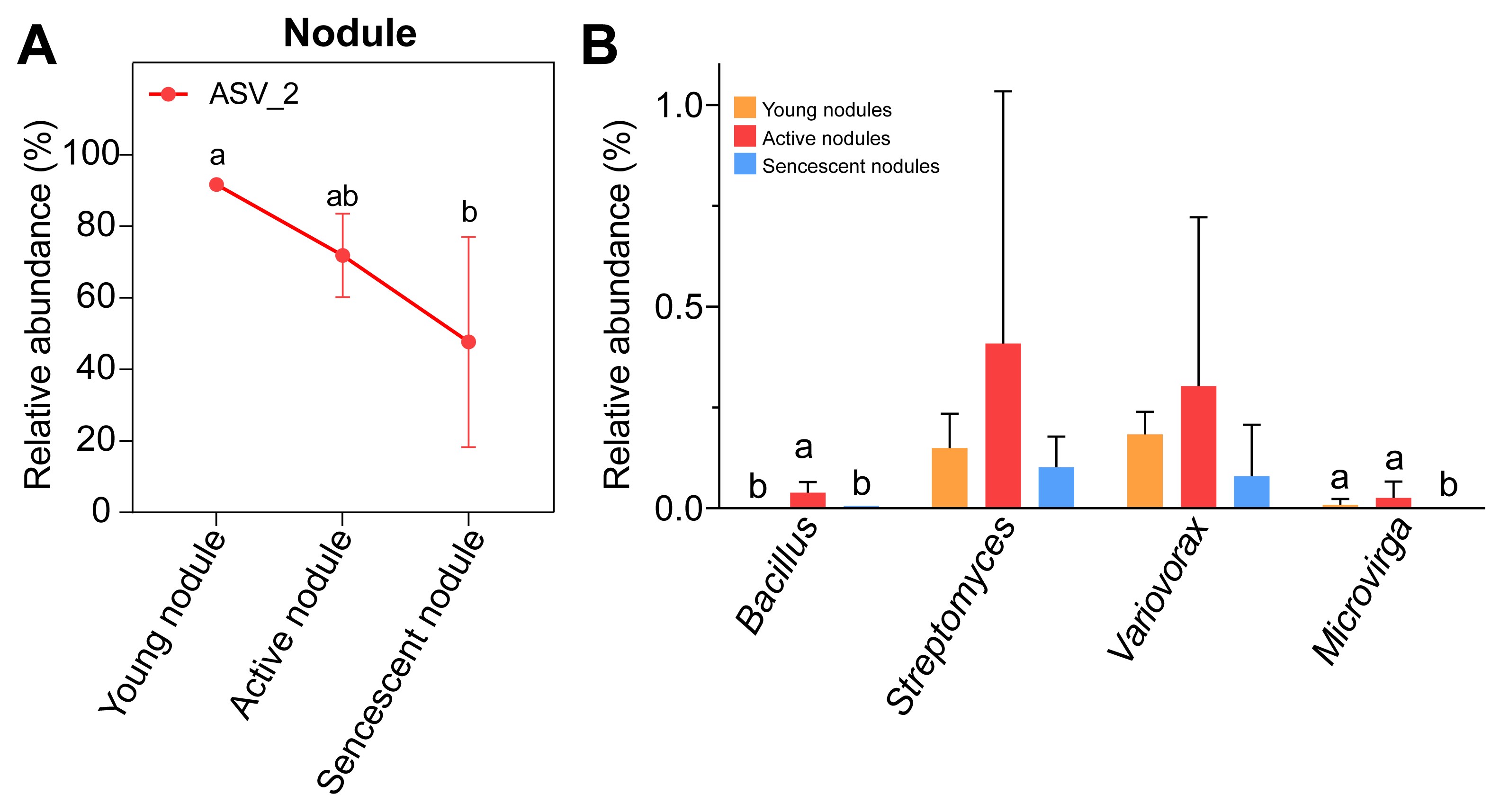

Supplement: Figure_S4_wrag087 [file figure_s4_wrag087.jpeg]

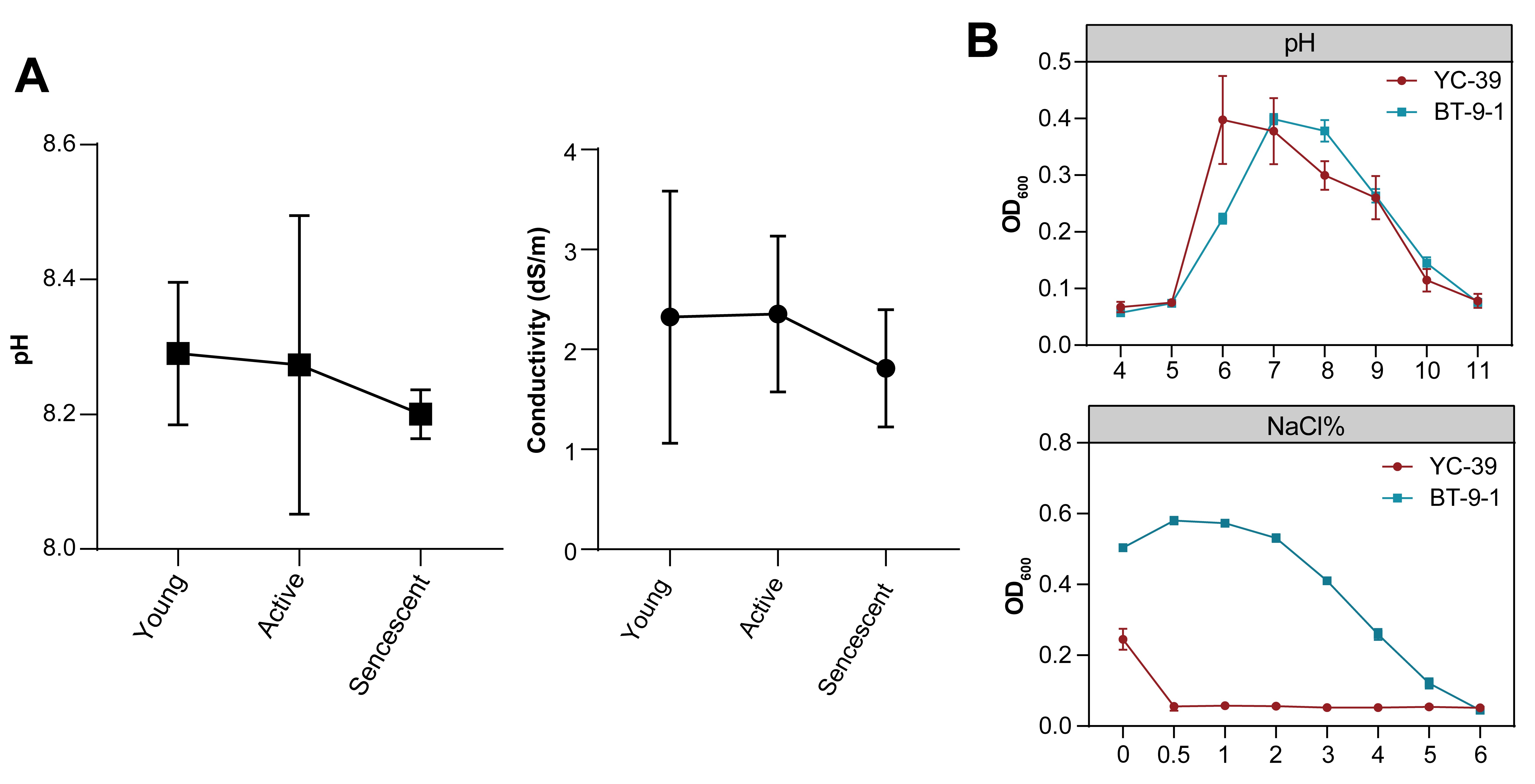

Supplement: Figure_S5_wrag087 [file figure_s5_wrag087.jpeg]

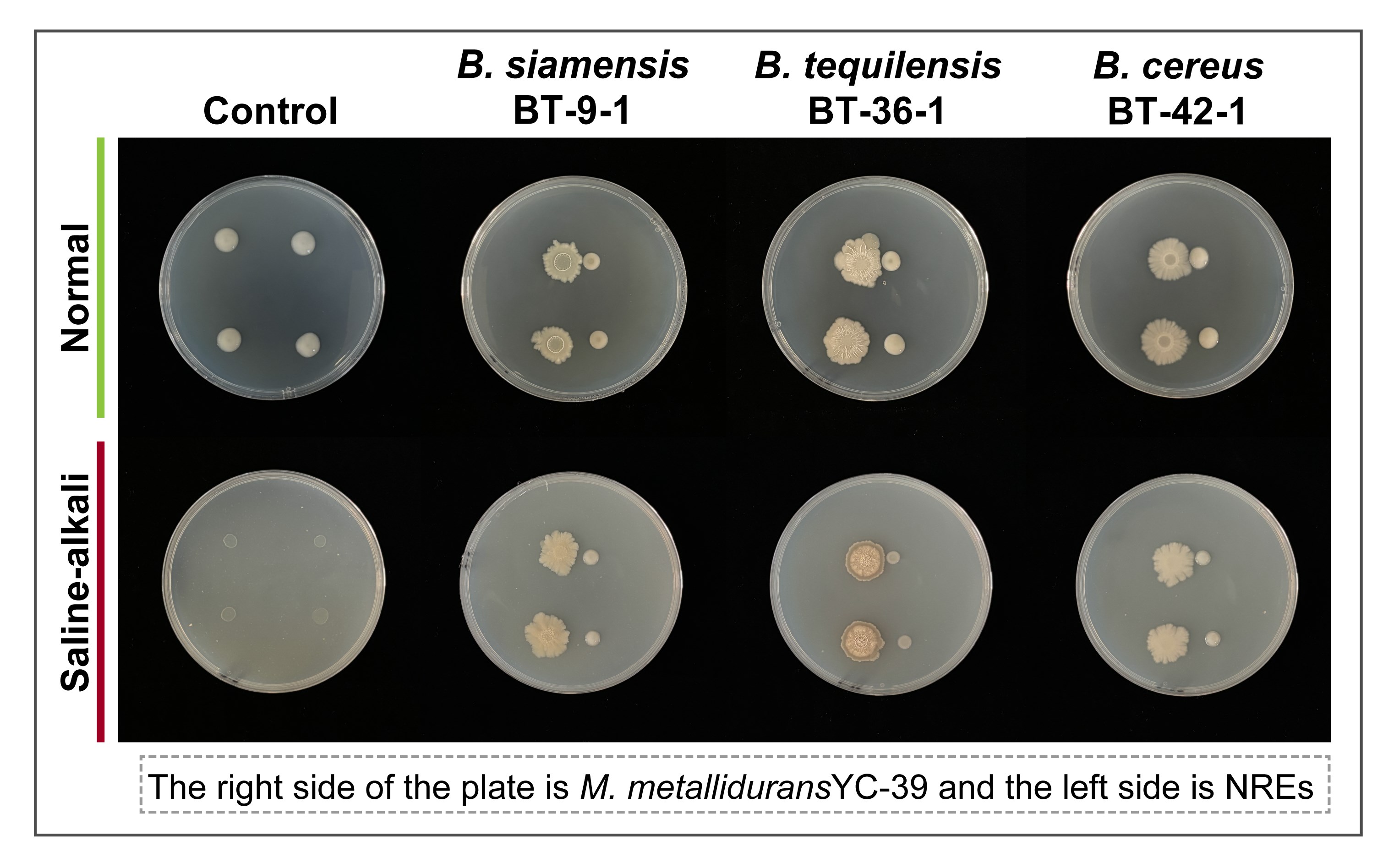

Supplement: Figure_S6_wrag087 [file figure_s6_wrag087.jpeg]

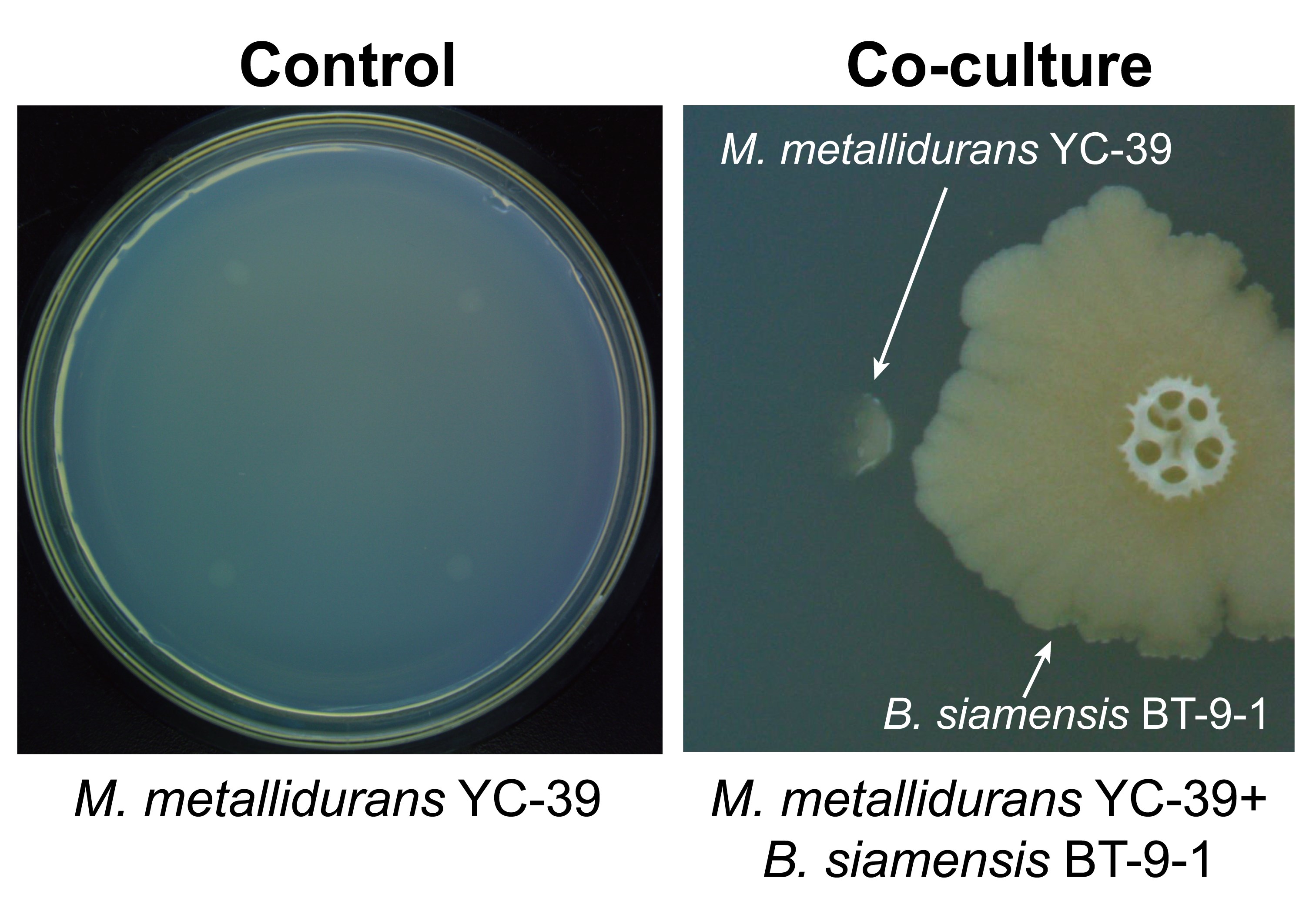

Supplement: Figure_S7_wrag087 [file figure_s7_wrag087.jpeg]

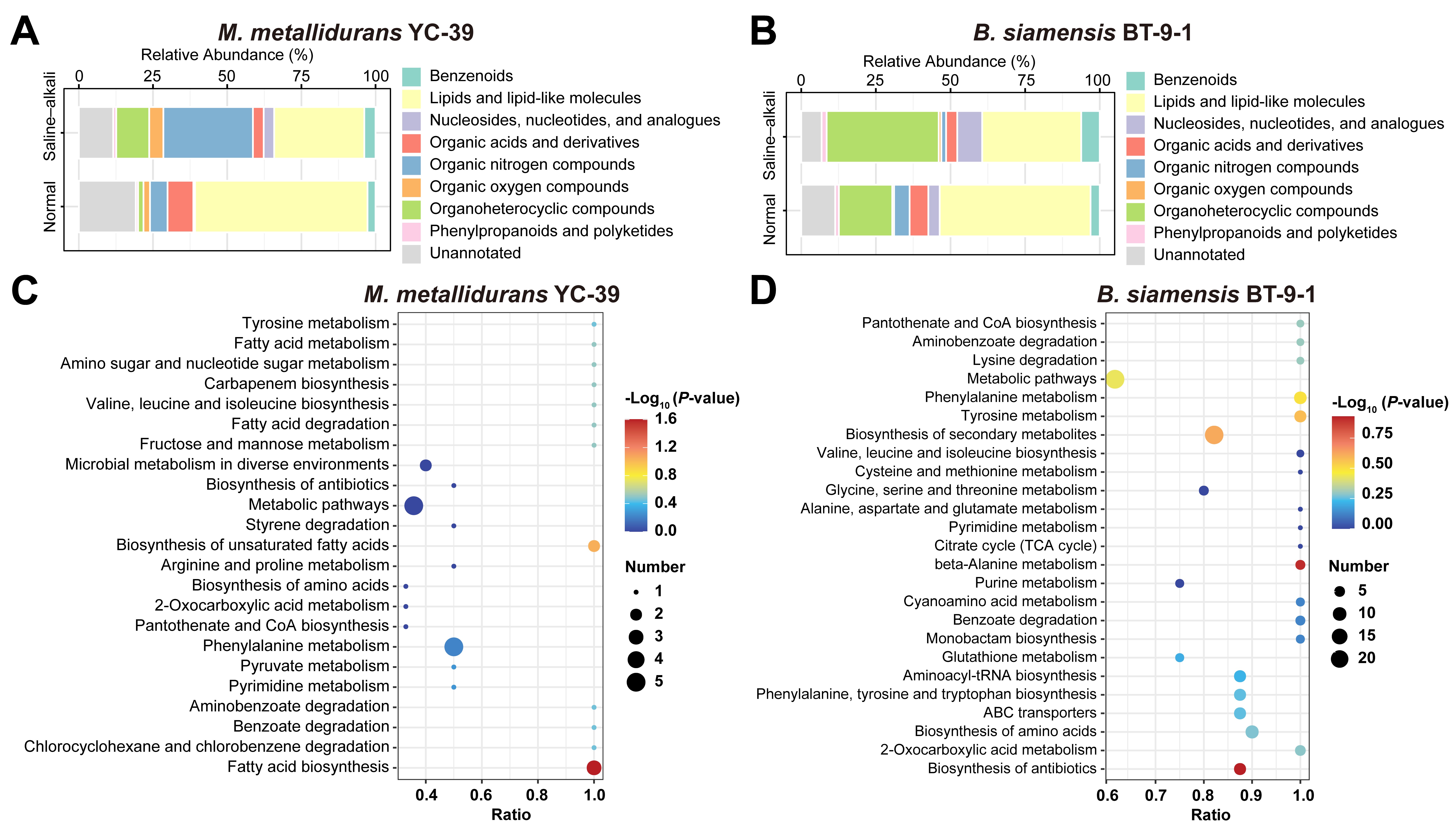

Supplement: Figure_S8_wrag087 [file figure_s8_wrag087.jpeg]

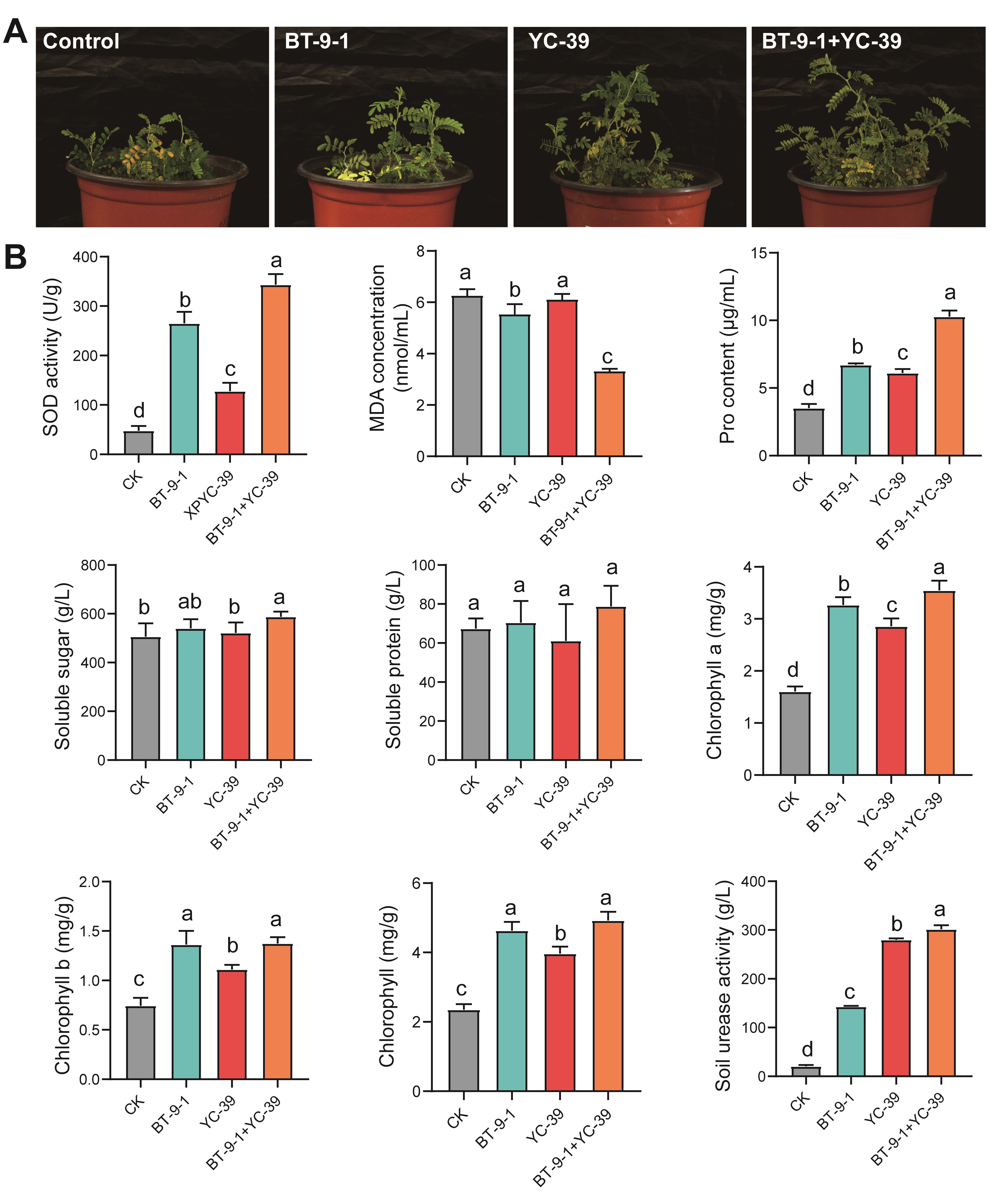

Supplement: Figure_S9_wrag087 [file figure_s9_wrag087.jpeg]

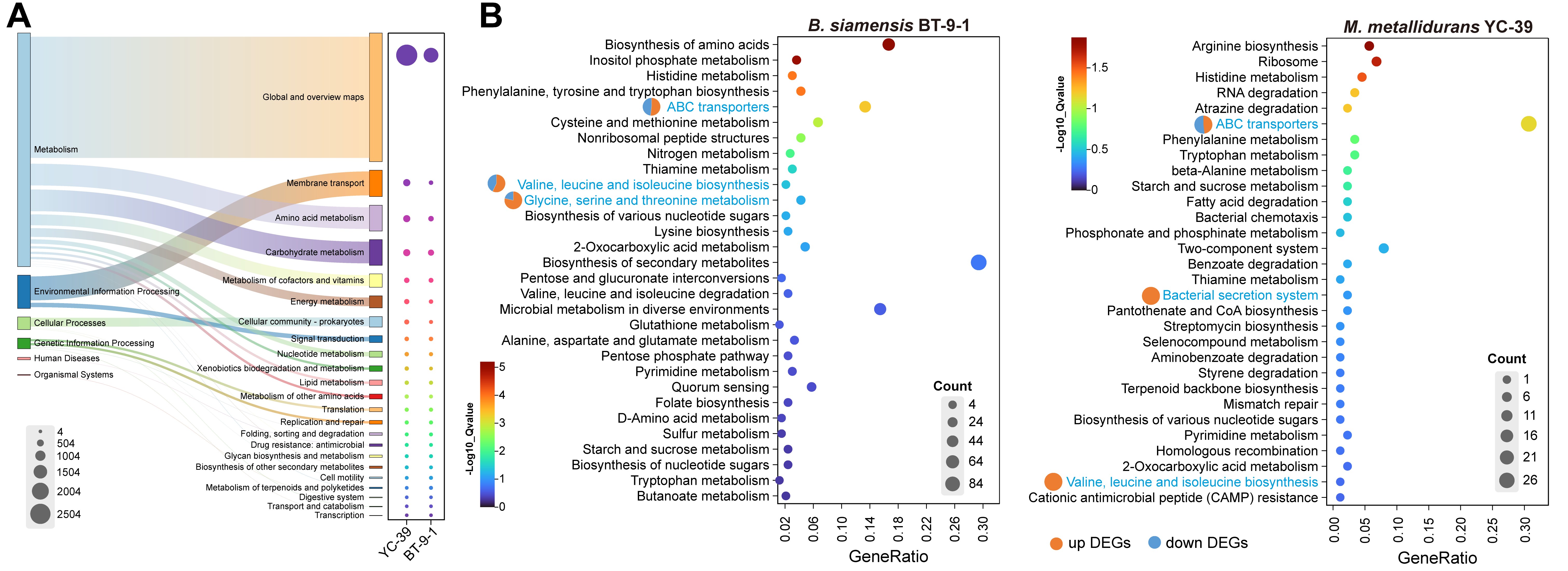

Supplement: Figure_S10_wrag087 [file figure_s10_wrag087.jpeg]
