# Supplementary material for: Bacillus populations restore amino acid metabolism in Mesorhizobium under saline–alkali stress to enhance nitrogen fixation efficiency
Source: ISME J. 2026 Apr 14;20(1):wrag087. doi: 10.1093/ismejo/wrag087 (PMC13155107; doi:10.1093/ismejo/wrag087)
Supplement: Supplemental_Material_1_wrag087 [file supplemental_material_1_wrag087.docx]

Running title: *Bacillus*-*Mesorhizobium* cross-feeding

*Bacillus* populations restore amino acid metabolism in *Mesorhizobium* under saline‒alkali stress to enhance nitrogen fixation efficiency

Jiamin Ai ^a, b^, Mingxia Ren ^b^, Ziwei Hao ^b^, Yuru Zhai ^a^, Entao Wang ^c^, Zhenshan Deng ^b,^*, Zhefei Li ^a,^*

^a^ State Key Laboratory for Crop Stress Resistance and High-Efficiency Production, Shaanxi Key Laboratory of Agricultural and Environmental Microbiology, College of Life Sciences, Northwest A&F University, Yangling, Shaanxi 712100, China

^b^ College of Life Sciences, Yan’an University, Yan’an 716000, China

^c^ Departamento de Microbiología, Escuela Nacional de Ciencias Biológicas, Instituto Politécnico Nacional, 11340, Cd. México, Mexico

***Correspondence:**

**Prof. Zhefei Li** (lizhefei@hotmail.com)

College of Life Sciences, Northwest A&F University, No. 3 Taicheng Road, Yangling District, Xianyang, Shaanxi Province, 712100, China

**Prof. Zhenshan Deng** (zhenshandeng214@163.com)

College of Life Sciences, Yan’an University, No. 580 Shengdi Road, Baota District, Yan’an, Shaanxi Province, 716000, China

Materials and methods

Isolation and characterization of symbiotic and NREs bacteria

All strains used in this experiment were non-rhizobial endophytes (NREs) of *S. davidii*, preserved in the laboratory. The isolation method of endophytic bacteria is as follows: the nodules were disinfected according to the method described by Sánchez-Cruz et al. [4]. The method is briefly described as follows: After 10 washes using sterile water, nodules were surface sterilized using 70% ethanol (5 min) and 2% sodium hypochlorite (10 min). The sterilized nodules were crushed and streaked on plates of LB (tryptone 10 g, yeast extract 5 g, NaCl 10 g, distilled water 1 L, pH 7.0-7.2) and yeast mannitol agar plates (YMA: yeast extract 3.0 g, mannitol 10 g, K_2_HPO_4_ 0.5 g, MgSO_4_·12H_2_O 0.2 g, NaCl 0.1 g, agar 18 g, distilled water 1 L, pH 7.0-7.2) [5]. The inoculated plates were incubated at 28°C for 5-7 days, and single colonies with distinct morphologies were picked up for purification by repeatedly streaking on YMA plates. All purified isolates were stored in 30% glycerol (v/v) at -80°C. To identify the isolates, each isolate was cultured for 2 days in 5 mL of YM broth with shaking at 180 rpm and 28°C for genomic DNA extraction using a Bacterial Genomic DNA Extraction Kit (D1600; Solarbio Science & Technology Co., Ltd., Beijing, China). The genomic DNA was amplified by PCR with primers of 27F (5´-AGAGTTTGATCCTGGCTCAG-3´) and 1429R (5´-GGTTACCTTGTTACGACTT-3´) [6], targeting the 16S rRNA gene. The PCR products were sequenced by Sangon Biotech Co., Ltd (Shanghai, China). The obtained sequences were submitted to BLAST analysis to find the most related homologous sequences. Phylogenetic trees were constructed using MEGA v5 with the neighbor-joining (NJ) method [7] to determine the species affiliation of the isolates. To confirm the presence of these bacterial isolates in the root nodules, similar analysis was performed based on the 16S rRNA sequences of the isolates and gene sequences of amplicon sequence variants (ASVs) (same genus as isolates). Isolates with V3-V4 region sequences matching ASVs with more than 97% similarity indicate that this species can be considered representative of the ASV [8]. The 16S rRNA sequences of isolates were uploaded to NCBI GenBank accession numbers PV942671-PV942678.

DNA extraction and sequencing

Total DNA was extracted separately from 0.5 g of samples (fresh nodules, rhizosphere, and bulk soil) using a TIANamp Soil DNA Kit (TIANGEN Biotech, Beijing, China) according to the manufacturer’s instructions. Before DNA extraction, the nodules were surface-sterilized and decontaminated to eliminate surface DNA, as described [1]. The V3-V4 region of the bacterial 16S rRNA genes were amplified using the barcoded primers 341F (5´-CCTAYGGGRBGCASCAG-3´) and 806R (5´-GGACTACNNGGGTATCTAAT-3´). The purified PCR products were sequenced on the Illumina NovaSeq platform at Novogene Bioinformatics Technology Co., Ltd (Beijing, China). Raw tags were filtered under specific conditions to obtain high-quality clean tags, following the QIIME 2 quality-controlled process [2]. The tags were compared to the SILVA reference database (v138) using UCHIME algorithm to remove chimeral sequences [3]. Before further analysis, all reads assigned to chloroplasts and mitochondria were discarded from the datasets. Sequences with ≥ 99% similarity were assigned to the same ASVs.

Exopolysaccharides (EPS) production and biofilm formation

For EPS extraction, a qualitative volume (1 mL) of *M. metallidurans* YC-39 and/or *B. siamensis* BT-9-1 (OD_600_ = 1) was inoculated in 100 mL YMB or saline‒alkali YMB (pH = 8.5, NaCl% = 0.5%). The cells were cultured at 28°C, 180 rpm. The mixture was then centrifuged at 10,000 rpm at 4°C for 10 minutes. The supernatant (5 mL) was transferred to a new centrifuge tube, and EPS precipitation was performed using 96% (v/v) cold ethanol at a volume ratio of 1: 4 (supernatant to ethanol). EPS was collected by centrifugation at 11,000 rpm for 30 min at 4°C and washed three times with 100% ethanol. After ethanol evaporation, the EPS was dissolved in ddH₂O, and the total EPS content was determined using the phenol-sulfuric acid method [9].

To observe the cocultured or monocultured pellicle biofilm formation, a 96-well microplate was employed, in which equal volume (1 μL) of *M. metallidurans* YC-39 and/or *B. siamensis* BT-9-1 (OD_600_ = 1) were inoculated in 100 μL YMB or saline‒alkali YMB (pH = 8.5, NaCl% = 0.5%). The inoculated plate was statically incubated at 28°C for 24 h. After 24 h of incubation, the planktonic cells were removed from each well, and the plates were rinsed 3 times with physiological saline. Excess water was removed, and the plate was inverted to dry in a sterile environment. Finally, pellicle biofilm formation was quantified using crystal violet staining followed by 30% glacial acetic acid, as described by Sabaeifard et al. [10].

Effects of metabolites on growth of strains

By comparing the interaction between different NREs and rhizobium on YMA plates under normal and saline‒alkali conditions, it was observed that *B. siamensis* BT-9-1 and *M. metallidurans* YC-39 exhibited significant interaction. To further investigate the effects of *B. siamensis* BT-9-1 metabolites on the growth of *M. metallidurans* YC-39 under different culture conditions, a growth curve of *M. metallidurans* YC-39 was constructed. *B. siamensis* BT-9-1 was cultured at 28°C with shaking (180 rpm) in YMB medium. After 2 days of incubation, the cultures were centrifuged, and the supernatants were filter-sterilized to obtain bacterial metabolites [11]. *M. metallidurans* YC-39 (100 μL, OD_600_ = 0.1) was inoculated into 10 mL of the metabolites from *B. siamensis* BT-9-1. The mixture was then incubated at 28°C with shaking (180 rpm) for an additional 54 h. The optical density (OD₆₀₀) was measured spectrophotometrically every 3 h to monitor growth. Cultures without metabolite addition were included as controls.

Metabolome analysis

To elucidate the mechanism of cooperation, the metabolome under coculture conditions was further analyzed. *M. metallidurans* YC-39 and *B. siamensis* BT-9-1 were cocultured on YMA and saline‒alkali YMA for 5 days, after which the colonies were collected into 15 mL sterile centrifuge tubes. The bacteria were rinsed 3 times with 5 mL of physiological saline, after which the excess water was removed. The collected precipitate was frozen in liquid nitrogen for 10 min and stored at -80℃.

The samples were placed in EP tubes and resuspended in prechilled 80% methanol by vortexing. Afterwards, the samples melted on ice and whirled for 30 s. After sonification for 6 min, they were centrifuged at 5,000 rpm, and 4°C for 1 min. The supernatant was freeze-dried and dissolved in 10% methanol. Then, the samples were sent to Novogene Bioinformatics Technology Co., Ltd (Beijing, China) for nontargeted metabolomics analysis using a Vanquish UHPLC system (Thermo Fisher) coupled with an Orbitrap Q Exactive HF-X mass spectrometer (Thermo Fisher). Samples were injected into a Hypersil GOLD column (C18) at a flow rate of 0.2 mL·min^-1^. In positive polarity mode, the eluents consisted of 0.1% formic acid (eluent A) and methanol (eluent B). For the negative polarity mode, 5 mM ammonium acetate (eluent A) and methanol (eluent B) were used. The mass spectrometer conditions included a spray voltage of 3.5 kV, a capillary temperature of 320°C, a sheath gas flow rate of 35 psi, and an auxiliary gas flow rate of 10 arb, with operation in both positive and negative polarity modes [12, 13].

The raw data files generated via UHPLC-MS/MS were processed using Compound Discoverer 3.1 (CD3.1; Thermo Fisher) to perform peak alignment, peak picking, and quantitation for each metabolite. The main parameters were as follows: retention time tolerance, 0.2 minutes; actual mass tolerance, 5 ppm; signal intensity tolerance, 30%; signal/noise ratio, 3; and minimum intensity. Peaks intensities were normalized to the total spectral intensity. The normalized data were used to predict the molecular formula based on additive ions, molecular ion peaks and fragment ions. Peaks were subsequently matched with the mzCloud (https://www.mzcloud.org/), mzVault and MassList databases to obtain accurate qualitative and relative quantitative results. Statistical analyses were performed using statistical software R (R version R4.3.2), Python (Python 3.10.7 version) and CentOS (CentOS release 6.6). When data were not normally distributed, normal transformations were attempted using the area normalization method.

These metabolites were annotated using the KEGG database (https://www.genome.jp/kegg/pathway.html), HMDB database (https://hmdb.ca/ metabolites) and LIPIDMaps database (http://www.lipidmaps.org/). We applied univariate analysis (t-test) to calculate the statistical significance (*P* value). The metabolites with a VIP > 1 and a *P* value < 0.05 and a fold change ≥ 2 or FC ≤ 0.5 were considered to be differential metabolites. Volcano plots were used to filter metabolites of interest based on the log2(fold change) and -log10(*P* value) values of metabolites by ggplot2 in R language. The functions of these metabolites and metabolic pathways were studied using the KEGG database. Metabolic pathway enrichment of differential metabolites was performed; when ratio was satisfied by x/n > y/N, metabolic pathway was considered enriched, and when *P* value of metabolic pathway was < 0.05, metabolic pathway was considered significantly enriched.

Detection of gene expression by qPT-PCR

The strains *M. metallidurans* YC-39 and *B. siamensis* BT-9-1 were co-cultured on YMA and saline‒alkali YMA plates for 5 days. The colonies were collected into 1.5 mL RNase-free centrifuge tubes, washed three times with RNase-free water, and the excess water was removed. Total RNA was extracted from the bacteria using the TaKaRa MiniBEST Universal RNA Extraction Kit (Takara Bio, France) according to the manufacturer's instructions. The isolated RNAs were reverse-transcribed into single-stranded complementary DNA (cDNA) using the PrimeScript RT reagent kit with a genomic DNA (gDNA) eraser (Takara Bio, France). Real-time PCR was performed on an ABI StepOnePlus Real-Time PCR System (Thermo Fisher Scientific) under the following conditions: cDNA was denatured for 30 s at 95°C, followed by 40 cycles of 5 s at 95°C and 30 s at 60°C. Primers were designed using Primer 5 based on the whole genomes of *M. metallidurans* YC-39 (JAOWPS000000000) and *Bacillus siamensis* KCTC 13613 (GCA_000262045.1), the latter being the most closely related strain to *B. siamensis* BT-9-1. Primer sequences are listed in Tables S3and S4. The transcript levels of *ilvA*, *ilvC*, *ilvD*, *ilvE*, *ilvH*, *leuA*, *leuB*, *leuC*, and *leuD* were evaluated by qRT-PCR using a TB Green Premix Ex Taq (Tli RNaseH Plus, ROX plus) (Takara Bio, France). The 16S rDNA was used as an internal control. The relative expression of target genes was calculated using the 2^-ΔΔCT^ method [14].

Design of simulated salt-alkaline conditions

*S. davidii* seeds collected from the sampling site were surface-sterilized and pre-germinated on water agar (0.4%) as described [15]. The germinated seeds were planted in pots (10 × 10 cm, 4 seeds per pot) containing sterilized vermiculite and cultured for 3 months in a greenhouse at 25°C under a 16 h light/8 h dark cycle. Three inoculation treatments were applied: 24 plants (six pots, four seeds per pot) were inoculated with 2 mL of *M. metallidurans* YC-39, *B. siamensis* BT-9-1, or a mixture of *M. metallidurans* YC-39 and *B. siamensis* BT-9-1 (1 : 1 by volume) three times, once at the beginning of each month. The control plants were watered with sterile water. After 3 months, 6 pots (3 replicates) for each inoculation treatment were subjected to salt-alkaline stress by adding a sterile salt-alkaline solution (sterile water with 0.5% NaCl, pH 8.5). The salt-alkaline solution was supplied via root irrigation (500 mL), and each pot was divided into three irrigation events every 2 days [16, 17]. The amount of evaporated water was determined by weighing the pots daily in the morning and evening, and the lost water was replenished with distilled water.

The malondialdehyde (MDA), superoxide dismutase (SOD), proline content (Pro), soluble sugar (SS), soluble protein (SP), chlorophyll a, chlorophyll b, and total chlorophyll contents were measured with the Malondialdehyde (MDA) Test Kit (Beijing Solarbio Science&Technology Co., Ltd, China), Superoxide Dismutase (SOD) Activity Test Kit (Beijing Solarbio Science&Technology Co., Ltd, China), Plant Soluble Sugar Content Assay Kit (Beijing Solarbio Science&Technology Co., Ltd, China), BCA Protein Concentration Measurement Kit (Biosharp, China) and Plant Chlorophyll Content Assay Kit (Beijing Solarbio Science&Technology Co., Ltd, China), respectively, according to the manufacturer’s protocol.

Effects of differential metabolites on the growth of rhizobia

The impact of differential metabolites ((2S)-Isopropylmalate and succinic acid) on the growth of *M. metallidurans* YC-39 was systematically evaluated. Standard materials of (2S)-Isopropylmalate (CAS: 3237-44-3, molecular weight: 176.17, AR), and succinic acid (CAS: 110-15-6, molecular weight: 118.09, AR) were purchased from Shanghai Aladdin Biochemical Technology Co., Ltd. Each compound was dissolved in sterile water to prepare a 10 mM stock solution. Bacterial cultures were initially inoculated in YMB medium and incubated at 170 rpm and 28°C for four days. The cultures were then adjusted to an optical density (OD) of 1.0 using sterile water. A 100 µL aliquot of the bacterial suspension was transferred to 10 mL of saline‒alkaline YMB medium. The two metabolites were added to achieve final concentrations of 25, 50, 100, and 200 μM. The bacteria were cultured at 28°C at 170 rpm for 4 days. Subsequently, the cultures were diluted 100-fold, and 30 µL of the diluted solution was plated onto YMA agar plates for colony-forming unit (CFU) enumeration.

Nodulation assay

Soil was collected from the sampling site for a nodulation test in the greenhouse. Surface-sterilized *S. davidii* seeds were planted in 10 × 10 cm pots. Different treatment groups were inoculated with a 2 mL suspension of *M. metallidurans* YC-39, a mixture of *M. metallidurans* YC-39 and *B. siamensis* BT-9-1 (1:1, v/v), or a mixture of *M. metallidurans* YC-39 and metabolites (1 : 1, v/v). Bacterial suspensions were inoculated on the 0th, 30th, and 60th days of plant growth, for a total of 3 inoculations during the plant growth period. Plants were grown in a greenhouse at 25°C for 3 months under a 16 h light/8 h dark cycle. After inoculation and treatment, the number of nodules and nitrogenase activity per plant were measured. Nitrogenase activity in root nodules was determined using the acetylene reduction assay [18, 19]. Paraffin sections of root nodules were then prepared and stained with toluidine blue. The sections were subsequently observed under an optical microscope.

**Determination of nitrogenase activity and observation of nodule sections**

A total of 0.02 g of nodules was placed in a 25 mL glass bottle, and 200 μL of acetylene gas was injected. The bottle was sealed with a sealing film and incubated at 28°C for 2 hours. Subsequently, 100 μL of gas was extracted using a microinjection needle, and the ethylene content was measured by gas chromatography. Nitrogenase activity was calculated based on a standard curve generated from ethylene standards.

The nodules were fixed in FAA fixative at 4°C for 36 hours. Following fixation, the nodules were dehydrated through graded ethanol and embedded in paraffin. Paraffin-embedded nodules were sectioned longitudinally into 8 μm slices and stained with 0.05% toluidine blue for 5 minutes [20]. Finally, the sections were observed under light microscopy (LM).

References

1. Sharaf H, Rodrigues RR, Moon J *et al.* Unprecedented bacterial community richness in soybean nodules vary with cultivar and water status. *Microbiome*. 2019;**7**:63 https://doi.org/10.1186/s40168-019-0676-8

2. Caporaso JG, Kuczynski J, Stombaugh J *et al.* Qiime allows analysis of high-throughput community sequencing data. *Nature Methods.* 2010;**7**:335-6 https://doi.org/10.1038/nmeth.f.303

3. Haas BJ, Gevers D, Earl AM *et al.* Chimeric 16s rrna sequence formation and detection in sanger and 454-pyrosequenced pcr amplicons. *Genome research.* 2011;**21**:494-504 https://doi.org/10.1101/gr.112730.110

4. Sánchez-Cruz R, Tpia Vázquez I, Batista-García RA *et al.* Isolation and characterization of endophytes from nodules of *Mimosa pudica* with biotechnological potential. *Microbiological Research.* 2019;**218**:76-86 https://doi.org/10.1016/j.micres.2018.09.008

5. Graham PH. Selective medium for growth of *Rhizobium*. *Applied Microbiology.* 1969;**17**:769-70 https://doi.org/10.1128/aem.17.5.769-770.1969

6. Weisburg WG, Barns SM, Pelletier DA *et al.* 16s ribosomal DNA amplification for phylogenetic study. *Journal of Bacteriology*. 1991;**173**:697-703 https://doi.org/10.1128/jb.173.2.697-703.1991

7. Tamura K, Peterson D, Peterson N *et al.* Mega5: Molecular evolutionary genetics analysis using maximum likelihood, evolutionary distance, and maximum parsimony methods. *Molecular Biology and Evolution*. 2011;**28**:2731-9 https://doi.org/10.1093/molbev/msr121

8. Zhou Y, Yang Z, Liu J *et al.* Crop rotation and native microbiome inoculation restore soil capacity to suppress a root disease. *Nature Communications.* 2023;**14**:8126 https://doi.org/10.1038/s41467-023-43926-4

9. Albalasmeh AA, Berhe AA, Ghezzehei TA. A new method for rapid determination of carbohydrate and total carbon concentrations using uv spectrophotometry. *Carbohydrate Polymers*. 2013;**97**:253-61 https://doi.org/10.1016/j.carbpol.2013.04.072

10. Sabaeifard P, Abdi-Ali A, Soudi MR *et al.* Optimization of tetrazolium salt assay for *Pseudomonas aeruginosa* biofilm using microtiter plate method. *Journal of Microbiological Methods*. 2014;**105**:134-40 https://doi.org/10.1016/j.mimet.2014.07.024

11. Sun X, Xu Z, Xie J *et al.* *Bacillus velezensis* stimulates resident rhizosphere *Pseudomonas stutzeri* for plant health through metabolic interactions. *The ISME Journal*. 2022;**16**:774-87 https://doi.org/10.1038/s41396-021-01125-3

12. Sellick CA, Hansen R, Stephens GM *et al.* Metabolite extraction from suspension-cultured mammalian cells for global metabolite profiling. *Nature Protocols.* 2011;**6**:1241-9 https://doi.org/10.1038/nprot.2011.366

13. Yuan M, Breitkopf SB, Yang X *et al.* A positive/negative ion-switching, targeted mass spectrometry-based metabolomics platform for bodily fluids, cells, and fresh and fixed tissue. *Nature Protocols.* 2012;**7**:872-81 https://doi.org/10.1038/nprot.2012.024

14. Livak KJ, Schmittgen TD. Analysis of relative gene expression data using real-time quantitative PCR and the 2(-Delta Delta C(T)) Method. *Methods*. 2001;**25**:402-8 https://doi.org/10.1006/meth.2001.1262

15. Ai J, Yu T, Liu X *et al.* Seed associated microbiota and vertical transmission of bacterial communities from seed to nodule in *Sophora davidii*. *Plant and Soil*. 2023;**491**:285-302 https://doi.org/10.1007/s11104-023-06115-2

16. Peng Y-L, Gao Z-W, Gao Y *et al.* Eco-physiological characteristics of *Alfalfa* seedlings in response to various mixed salt-alkaline stresses. *Journal of Integrative Plant Biology*. 2008;**50**:29-39 https://doi.org/10.1111/j.1744-7909.2007.00607.x

17. Lv Y, Xu N, Ha M *et al.* *Bacillus cereus* enhances salt tolerance of cucumber seedlings by improving antioxidant metabolism and decreasing the ion toxicity. *Scientia Horticulturae*. 2024;**328**:112885 https://doi.org/10.1016/j.scienta.2024.112885

18. Montes-Luz B, Conrado AC, Ellingsen JK *et al.* Acetylene reduction assay: A measure of nitrogenase activity in plants and bacteria. *Current Protocols*. 2023;**3**:e766 https://doi.org/10.1002/cpz1.766

19. Fishbeck K, Evans HJ, Boersma LL. Measurement of nitrogenase activity of intact legume symbionts in situ using the acetylene reduction assay. *Agronomy Journal*. 1973;**65**:429-33 https://doi.org/10.2134/agronj1973.00021962006500030022x

20. Feng Z, Zhang L, Wu Y *et al.* The Rpf84 gene, encoding a ribosomal large subunit protein, RPL22, regulates symbiotic nodulation in *Robinia pseudoacacia*. *Planta*. 2019;**250**:1897-910 https://doi.org/10.1007/s00425-019-03267-3

Supplementary Tables and Figures:

**Table S1** Amino acid differential metabolic pathways and differential metabolites of *M. metallidurans* YC-39 under saline‒alkaline stress

**Table S2** Amino acid differential metabolic pathways and differential metabolites of *B. siamensis* BT-9-1 under saline‒alkaline stress

**Table S3** Primer sequences of *B. siamensis* BT-9-1

**Table S4** Primer sequences of *M. metallidurans* YC-39

**Figure S1.** Schematic diagram of sample collection (A). The schematic diagram of chemotaxis assay (B).

**Figure S2.** Microbial community composition in different compartments at three stages of nodule development. A. Principal coordinate analysis (PCoA) of microbial communities on the basis of Bray‒Curtis distances. Y: young stage; A: active stage; S: senescent stage; BS: bulk soil; R: rhizosphere soil; N: nodule. B. Taxonomic composition of bacterial communities at the phylum level across developmental stages. C. Bacterial phyla exhibiting significant changes in relative abundance during nodule development. Lowercase letters above the boxes indicate significant differences (*P* < 0.05) between the different treatments (one-way ANOVA). D. Bacterial genus with significant changes in relative abundance during nodule development. The bacterial genera with significant differences between groups were analyzed using ANOVA and Duncan’s multiple range test. Lowercase letters indicate significant differences (*P* < 0.05) between the different treatments. The relative abundance was log_10_ transformed.

**Figure S3.** The co-occurrence network analysis. A. The co-occurrence networks across different compartments. B. Topological parameters of co-occurrence networks in different samples.

**Figure S4.** Changes in relative abundance of core species. A. The relative abundance of ASV_2 (*Mesorhizobium*) in nodule samples throughout nodule development. B. Relative abundance of core genus in nodule samples throughout nodule development. The lowercase letters located above the bars indicate significant differences in relative abundance during the three stages of nodules development (*P* < 0.05) (one-way ANOVA). No significant differences are not marked.

**Figure S5.** Environmental factors of sampling sites and stress resistance test of strains. A. Soil pH, and conductivity at the sampling site. B. Strains tolerance to varying pH and NaCl concentrations.

**Figure S6.** Analysis of the interaction between rhizobia and non-symbiotic endophytes (NREs) under different culture conditions.

**Figure S7.** The interaction between *M. metallidurans* YC-39 and *B. siamensis* BT-9-1 under saline‒alkali stress at low inoculation concentration (OD_600_ = 0.5).

**Figure S8.** Metabolic profiling and pathway enrichment of *M. metallidurans* YC-39 and *B. siamensis* BT-9-1 under saline-alkali stress. Relative abundance of metabolite categories in YC-39 (A) and BT-9-1 (B) under normal and saline-alkali conditions. Bubble plots showing the differentially enriched metabolic pathways for YC-39 (C) and BT-9-1 (D). The x-axis represents the enrichment ratio, and the bubble size indicates the number of metabolites involved. The color scale represents the significance level defined by -log_10_ (*P*-value). The enrichment significance was determined by calculating raw *P* values using the hypergeometric test, followed by adjustment via the Benjamini–Hochberg false discovery rate (FDR) correction method.

**Figure S9.** The consortium promoted *S. davidii* plant growth and alleviated saline‒alkali stress. Lowercase letters above the bars indicate significant differences (*P* < 0.05) between the different treatments (one-way ANOVA).

**Figure S10.** Transcriptome analysis results. A. The KEGG classification of genes. B. The KEGG enrichment pathway of *M. metallidurans* YC-39 and *B. siamensis* BT-9-1 under saline‒alkali stress.
